# Tapering and Peaking in the Weight Lifting Sports: A Systematic Review of Athletes’ Self-Reported Strategies

**DOI:** 10.1007/s40279-026-02500-w

**Published:** 2026-08-18

**Authors:** Paul W. Winwood, S. Kyle Travis, Matthew J. Barnes, Justin W. L. Keogh, Hayden J. Pritchard

**Affiliations:** 1 Department of Sport and Fitness, Faculty of Health, Education and Environment, Toi Ohomai Institute of Technology, Tauranga, New Zealand; 2 Te Huataki Waiora School of Health, The University of Waikato, Hamilton, New Zealand; 3 School of Health Sciences, Department of Allied Health Professions, Liberty University, Lynchburg, VA USA; 4 Science, Medicine, Innovation & Technology (SMIT) Laboratory, nøsisX, Elizabethton, TN USA; 5 School of Sport, Exercise and Nutrition, College of Health, Massey University, Palmerston North, New Zealand; 6 Faculty of Health Sciences and Medicine, Bond University, Gold Coast, Australia; 7 Sports Performance Research Centre New Zealand, Auckland University of Technology, Auckland, New Zealand; 8 Department of Physiotherapy, Kasturba Medical College Mangalore, Manipal Academy of Higher Education, Manipal, India; 9 HJP Method Coaching and Consulting, Palmerston North, New Zealand

## Abstract

**Background:**

Tapering, the reduction of training load before competition, is widely recognized as an important strategy for optimizing performance. While tapering practices have been well documented in endurance sports, comparatively less is known about their application in weight lifting sports such as CrossFit, Highland Games, powerlifting, strongman and weightlifting.

**Objectives:**

To systematically review the available evidence on athletes self-reported tapering practices in weight lifting sports in accordance with PRISMA guidelines.

**Methods:**

An electronic search was performed using PubMed, Embase, SPORTDiscus and Scopus for studies that examined athletes self-reported tapering practices in weight lifting sports. Eligible studies included peer-reviewed journal articles only, with no limit placed on date or language of publication. The methodological quality of included studies was assessed using the AXIS critical appraisal tool for cross-sectional studies.

**Results:**

All seven studies were rated high quality (≥ 80% score, low risk of bias); five used surveys and two powerlifting studies used interviews. Tapering prevalence remained consistently high across sports, with little variation by competition level, age, or bodyweight class. Step tapers were most commonly reported, with linear and exponential models less frequent. Taper length typically ranged from five to nine days, although longer durations were reported in powerlifting. Weightlifting and CrossFit athletes most often reported formal coaching, while self-coaching was more common in strongman and Highland Games; female athletes generally reported greater coaching access than males. Training cessation periods were between 1.5 and 4 days. Athletes typically reached peak training volume 4–6 weeks and peak intensity 2–3 weeks before competition, with taper phases characterized by a reduction in training volume of ~ 40–45%. Deadlifts were typically last performed 6–8 days before competition, squats 5–6.5 days, and the bench press 3–5 days. The most consistent taper adjustment was reduced training duration, with CrossFit emphasizing aerobic and gymnastics work, Highland Games and strongman prioritizing power and strength, and weightlifting focusing on strength, power, and technical work. Additional tapering strategies included nutrition and recovery methods. Athletes reported tapering to enhance recovery, performance, and psychological readiness, while ineffective tapering was linked to errors in load management and contextual stressors such as work, travel, and nutrition.

**Conclusion:**

Tapering in weight lifting sports is typically short and structured, with step tapers, reduced training volume, and lift-specific timing most common, but effectiveness depends on managing both training load and contextual stressors. These findings underscore the multifactorial nature of tapering and highlight the need for evidence based, sport specific recommendations.

## Key Points

**Table:** 

Athletes typically employed short tapers of 5–9 days, with step tapers most common and training volume reduced by ~40–45%. This contrasts with endurance sports, where longer taper durations are often reported underscoring discipline-specific differences.
Tapering differed by exercise and sport. Deadlifts (6–8 days), squats (5–6.5 days), and bench press (3–5 days) were performed at distinct intervals before competition, with sport-specific differences in training emphasis also observed.
Athletes tapered to optimize recovery, performance, and psychological readiness, but missteps often stemmed from poor load management or external stressors such as work, travel, and nutrition, highlighting the need for individualized, evidence-based guidelines.

## Introduction

Maximizing performance at the time of competition is a central goal of training periodization. Athletes spend months or years in training, with the outcome ultimately determined by performance on the day of the event. Peaking represents the final phase of training, during which training is manipulated to maximise performance at a specific time point [1]. While improving performance is a general aim of training, peaking refers specifically to the timing of these adaptations to coincide with competition. Tapering is one strategy used within this phase and has been defined as “a progressive nonlinear reduction of the training load during a variable period of time, in an attempt to reduce the physiological and psychological stress of daily training and optimize sports performance” [2]. Although often used interchangeably, peaking and tapering are distinct; a peak may or may not involve a taper. For example, an athlete may maintain regular training and then reduce or cease training in the days immediately prior to competition, which falls outside the definition of tapering but may still enhance performance [3]. Recognizing this distinction is particularly important in weight lifting sports, where preparation methods often reflect tradition or individual preference rather than evidence-based practice [4].

Weight lifting sports such as CrossFit, powerlifting, strongman, and weightlifting have grown in global popularity over the last decade [5]. This growth has been accompanied by greater visibility at international competitions and through social media. Despite this rise, much of the scientific literature on tapering and peaking has focused on endurance-based sports [1], leaving less clarity on how athletes in weight lifting sports should prepare to optimize competition performance. Experimental studies focused on powerlifting indicate that tapering is effective, reporting no decrements in performance and increases of 3.2–4.4% (14.1–27.7 kg) in powerlifting total across different peaking approaches [1]. These findings demonstrate that tapering and peaking are important phases of training for competition outcomes.

The fitness–fatigue model provides a useful framework for understanding how peaking strategies can enhance performance. It proposes that training produces two after-effects: fitness, the positive adaptations that improve performance, and fatigue, the negative consequences that suppress it. Fatigue effects are generally larger in magnitude but dissipate more rapidly, whereas fitness effects are smaller but longer lasting [6]. A taper, and related peaking methods, therefore aims to reduce accumulated fatigue while maintaining or reinforcing fitness adaptations, thereby maximizing performance. However, improvements in physiological readiness do not necessarily guarantee enhanced competition performance, as outcomes are also influenced by psychological factors, competition-day preparation, and contextual stressors [7, 8].

Commonly described approaches include the linear taper, the exponential taper (fast or slow decay), and the step taper [1]. In a linear taper, training load decreases at a consistent rate (e.g., ~ 20% each week for three weeks), whereas in a step taper, load is reduced by a fixed amount at once and maintained (e.g., 50% reduction in the final week). An exponential taper, with a fast decay, involves a large initial reduction that then levels off (e.g., 50% in week one, followed by smaller reductions) while, in contrast, a slow decay taper begins with minor reductions that become progressively larger as competition approaches (e.g., 10% in week one, 30% in week two, 50% in week three).

In recent years, research examining tapering and peaking strategies used by athletes in weight lifting sports has grown steadily. Following an early interview-based study of elite New Zealand powerlifters in 2016 [4], several investigations have since employed surveys or interviews to explore how weight lifting athletes taper across different disciplines, ranging from strongman [9] to CrossFit [10]. To date, however, no synthesis has examined the commonalities and differences in the tapering and peaking practices of weight lifting athletes.

Although these sports differ in structure and competition demands, they share a common reliance on outcomes being determined by performances involving lifting of heavy external loads. Therefore, for the purpose of this review, weight lifting sports are defined as ‘sports in which the central focus is the lifting, carrying, or throwing of weighted implements (e.g., barbells, stones, logs, and other heavy objects) as a measure of power, endurance, and maximal or functional strength.’

The aim of this systematic review was to descriptively synthesise survey- and interview-based research to better understand how weight lifting athletes manage tapering and peaking practices in real-world settings, including the training undertaken in the lead-up to competition. The review focuses on describing athlete-reported practices rather than determining the effectiveness of specific tapering strategies. Given the limited experimental literature in weight lifting sports, self-reported data provide important insight into real-world tapering practices, including decision-making processes (e.g., coach-led vs. self-directed approaches) and contextual factors (e.g., work, travel, nutrition, and recovery environment) that are not readily captured in controlled studies. It was hypothesised that athletes surveyed or interviewed in the included studies would report using tapering or peaking strategies before competition, typically involving reduced training volume and variable adjustments in training intensity.

## Methods

### Protocol and Reporting Standards

This systematic review was conducted in accordance with the Preferred Reporting Items for Systematic Reviews and Meta-Analyses (PRISMA) guidelines [11]. The review protocol was preregistered on the Open Science Framework (OSF) prior to initiation of the search procedures (https://doi.org/10.17605/OSF.IO/XQ64B).

### Search Strategy and Eligibility Criteria

Peer-reviewed literature was identified through searches of PubMed, Embase, Scopus, and SPORTDiscus on 30 July 2025. Search terms were developed around two core concept groups: (i) tapering-related terms (competition preparation, peaking, training cessation, training decrease, training reduction, taper) and (ii) sport-related terms (CrossFit, cross fit, Highland game, Highland games, weightlifting, power lift, power lifting, powerlifting, resistance training, strength sport, strength training, strongman, strong man, weight lift, weight lifting). Boolean operators (AND/OR) and database-specific syntax were applied to combine these terms.

For example, the PubMed search strategy was as follows: ("Tapering"[Text Word] OR "Taper"[Text Word] OR "Training reduction"[Text Word] OR "Training cessation"[Text Word] OR "Unloading"[Text Word] OR "Peaking"[Text Word] OR "Training decrease"[Text Word] OR "Tapering"[MeSH Terms]) AND ("Weight Lifting"[MeSH Terms] OR weight lift*[Text Word] OR weightlift*[Text Word] OR power lift*[Text Word] OR powerlift*[Text Word] OR strongman[Text Word] OR "strong man"[Text Word] OR "Highland Game"[Text Word] OR "Highland Games"[Text Word] OR "Cross fit"[Text Word] OR CrossFit[Text Word]).

Studies were eligible for inclusion if they involved competitors in weight lifting sports (e.g., CrossFit, Highland Games, powerlifting, strongman and weightlifting) who self-reported tapering or peaking strategies through surveys or interviews. Studies were excluded if they focused on recreational or youth athletes, did not collect self-reported data, were case studies, or were not peer-reviewed. All included studies involved adult athlete populations (≥ 18 years). No restrictions were placed on year of publication or language.

### Study Selection and Data Extraction

Titles and abstracts were independently screened by two reviewers (PW and HP) to assess potential eligibility. Full-text versions of all articles deemed relevant were then assessed against the inclusion criteria. Reference lists of included studies were examined to identify additional records not captured through the initial database searches. Citation tracking was also performed in Google Scholar to locate further relevant records. Disagreements between reviewers were resolved through discussion until consensus was reached.

Data were extracted by one reviewer and checked by a second using a standardized format. Extracted information included (i) study characteristics (sport type, country, year, method [survey or interview], sample size, demographics, and competitive level), (ii) tapering practices (use of taper [yes/no], taper length, taper type [e.g. step, linear, exponential], timing of final sessions, and changes in training volume, intensity, frequency, and exercise selection), and (iii) taper rationale (reported reasons for tapering, decision-making approach [e.g. trial and error, by feel, coached], and additional strategies such as nutrition, mobility, or rest). For qualitative data, themes relating to perceived taper goals, lift-specific differences, and factors associated with poor or successful tapering were also extracted.

Findings from included studies were synthesized narratively. Proportions or frequencies were reported descriptively, and categorical data such as tapering strategies were grouped into common themes for consistency. Missing or incomplete data were noted, with no imputation undertaken.

### Study Quality

The methodological quality of included studies was assessed using the AXIS critical appraisal tool for cross-sectional studies [12]. Each item was scored according to AXIS guidelines, with higher totals reflecting stronger methodological quality. To ensure the scoring accurately reflected study quality, negatively framed items (questions 13 and 19) were inverted. Quality assessments were completed by one reviewer and checked by a second, with any disagreements resolved through discussion until consensus was reached. Overall scores were converted to percentages and rated as follows: ≥ 80% high quality (low risk of bias), 60–79% fair quality (moderate risk), and < 60% low quality (high risk).

## Results

### Literature Search

**Fig. 1:**
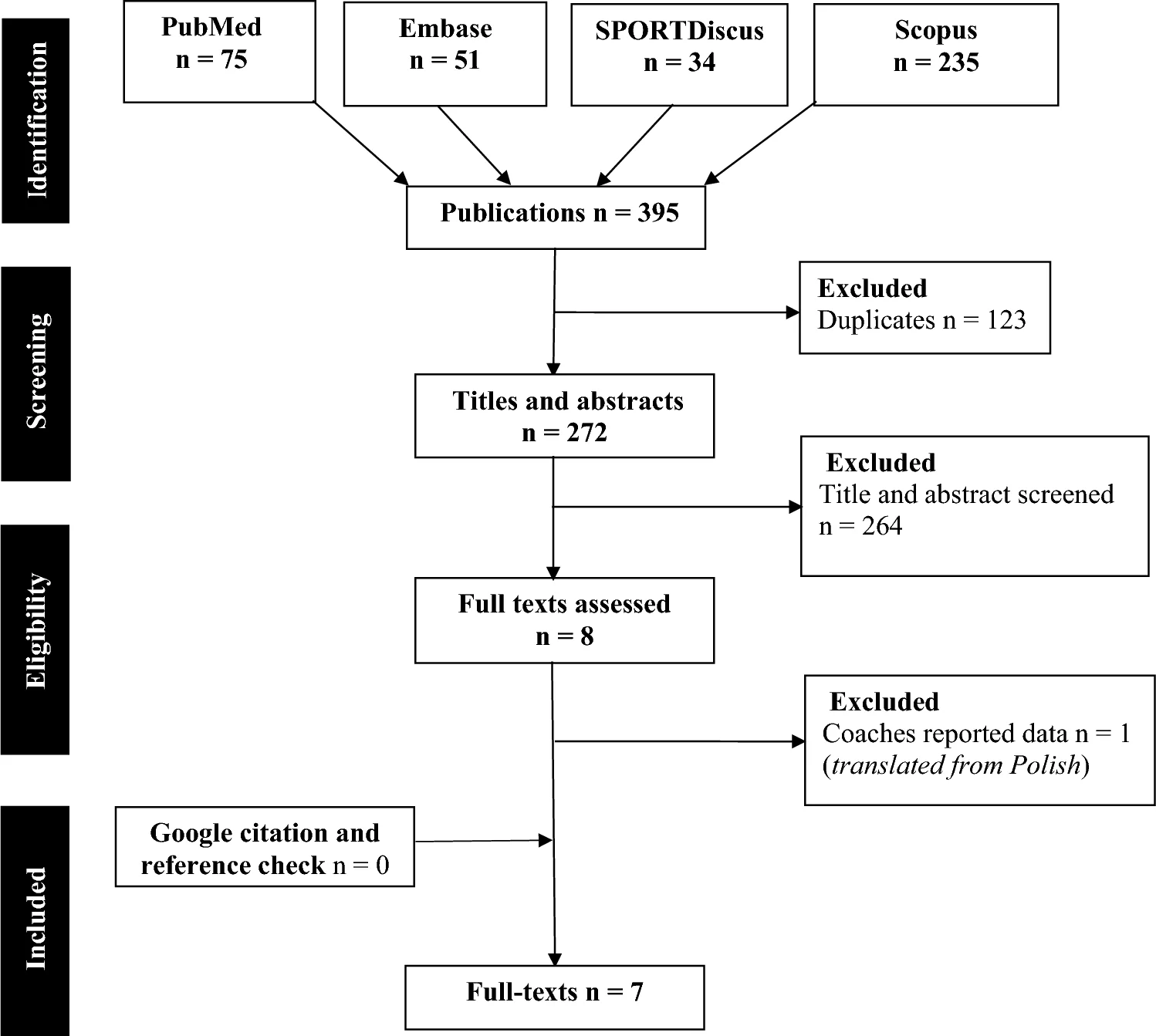
Flow chart of the article selection process

An examination of 395 titles (including 123 duplicates) revealed 272 potentially relevant full-text articles which were retrieved. After review of the titles and abstracts, a further 264 articles were excluded. The full text of eight studies were then reviewed. One study translated from Polish was excluded. Reference lists and forward citation searches of the seven included studies did not identify any additional eligible studies. A pictorial representation of the search and screening process is provided in Fig. 1.

Of the seven eligible studies, three examined powerlifting [4, 13,14], and one study each investigated CrossFit [10], Highland Games [15], strongman [9] and weightlifting [16]. All studies presented data categorized by sex, while several also reported results by competitive standard [9, 13–16], age [9, 13], and bodyweight class [9, 13].

### Risk of Bias

The results of the risk of bias (ROB) assessment are displayed in Table 1. All seven included studies achieved a score ≥ 80% and were therefore considered to have a low risk of bias. Specifically, scores ranged from 16 (80%) to 18 (90%). Three studies achieved scores of 16.5 (82.5%) [9, 15,16] and one study scored 16 (80%) [10], while the highest score of 18 (90%) was recorded in powerlifting [14].

**Table 1:** Study quality scores obtained for the appraisal tool for cross sectional studies (AXIS)

	CrossFit	Highland Games	Powerlifting			Strongman	Weightlifting
	Pritchard et al. [8]	Winwood et al. [13]	Grgic and Mikulic [12]	Pritchard et al. [6]	Travis et al. [11]	Winwood et al. [7]	Winwood et al. [14]
1	1	1	1	1	1	1	1
2	1	1	1	1	1	1	1
3	1	0.5	1	1	0.5	0.5	0.5
4	1	1	1	1	1	1	1
5	1	1	1	1	1	1	1
6	0.5	0.5	1	1	0.5	0.5	0.5
7	0	0	0	0	0	0	0
8	1	1	1	1	1	1	1
9	1	1	1	0.5	1	1	1
10	1	1	1	1	1	1	1
11	1	1	1	1	1	1	1
12	1	1	1	1	1	1	1
13*	0	1	1	1	1	1	1
14	0	0	0	0	0	0	0
15	1	1	1	1	1	1	1
16	1	1	1	1	1	1	1
17	1	1	1	1	1	1	1
18	0.5	0.5	1	0.5	1	0.5	0.5
19*	1	1	1	1	1	1	1
20	1	1	1	1	1	1	1
Total Appraisal Score	16 (80%)	16.5 (82.5%)	18 (90%)	17 (85%)	17 (85%)	16.5 (82.5%)	16.5 (82.5%)
Study Quality	Good	Good	Good	Good	Good	Good	Good
Risk of Bias	Low risk	Low risk	Low risk	Low risk	Low risk	Low risk	Low risk

^*^Inverse scores applied as a positive response indicates poorer study quality

The studies provided clear aims and objectives and employed appropriate study designs. All seven studies adequately reported basic methodological aspects such as the justification of sample size, outcome measurement validity, and clarity of results. However, consistent limitations were identified. Several studies reported survey participation or completion rates; however, non-response reporting was inconsistent, and in some studies the total number of athletes who received the survey invitation could not be determined because recruitment occurred through indirect distribution via coaches, organisations, clubs, or online networks. In addition, the reporting of conflicts of interest and funding sources was not presented with the same level of detail or consistency across studies. Several studies provided partial clarity on issues such as the representativeness of the sample, scoring 0.5, indicating moderate rather than full compliance.

### Study Design and Athlete Demographics

A summary of the study design and athlete demographics is provided in Table 2. Of the seven studies included, five studies employed surveys [9,10, 13, 15,16] and two utilized interviews [4, 14]. Athlete demographics varied across the five weight lifting sports. Elite CrossFit athletes were younger (26.5 ± 3.6 y), shorter (167.1 ± 9.5 cm) and lighter (74.5 ± 12.7 kg) than athletes from the other weight lifting sports. Highland Games heavy event athletes were the oldest (40.8 ± 10.7 y), tallest (181.2 ± 9.5 cm) and reported the longest general training (18.8 ± 10.3 y) and sport-specific training (8.4 ± 7.3 y) backgrounds. Strongman athletes were also relatively large (178.1 ± 10.6 cm; 108.6 ± 27.9 kg) with an average age of 33.2 ± 8.0 y, and professionals reported the greatest training experience (16.8 ± 9.3 y general training, 8.8 ± 6.2 y sport-specific training). Weightlifters were younger (29.2 ± 8.7 y), shorter (172.5 ± 10.1 cm) and lighter (84.0 ± 17.2 kg) than strongman and Highland Games heavy event athletes, with 9.5 ± 7.5 y of general training and 4.7 ± 3.4 y of sport-specific training experience. Powerlifters were of similar age (≈28–29 y) but generally heavier (male 86–95 kg; female 64–80 kg), though data reporting was less consistent, particularly for height and training background.

**Table 2:** Summary of the included studies and athlete demographics

Study	Study Design	Athletes	Age (y)	Height (cm)	Body Mass (kg)	General Training Experience (y)	Sport Specific Training Experience (y)
CrossFit							
Pritchard et al. [8]	Survey	72 elite	26.5 ± 3.6	167.1 ± 9.5	74.5 ± 12.7	12.8 ± 6.5	5.4 ± 1.7
Pritchard et al. [8]	Survey	39 ♀ elite	27.3 ± 3.7	161.2 ± 26.6	63.9 ± 11.5	14.0 ± 7.2	5.5 ± 1.9
Pritchard et al. [8]	Survey	33 ♂ elite	25.7 ± 3.5	174.0 ± 7.5	86.6 ± 5.9	11.2 ± 5.2	5.3 ± 1.5
Highland Games							
Winwood et al. [13]	Survey	169	40.8 ± 10.7	181.2 ± 9.5	107.2 ± 23.0	18.8 ± 10.3	8.4 ± 7.3
Winwood et al. [13]	Survey	118 ♂	41.5 ± 10.4	185.0 ± 7.5	112.9 ± 21.8	20.2 ± 10.1	9.4 ± 8.0
Winwood et al. [13]	Survey	51 ♀	39.5 ± 11.3	172.1 ± 8.1	89.6 ± 22.2	15.5 ± 10.4	6.1 ± 4.8
Winwood et al. [13]	Survey	74 Local/Reg	37.4 ± 9.2	178.4 ± 8.6	105.9 ± 25.8	15.4 ± 9.8	4.5 ± 4.2
Winwood et al. [13]	Survey	22 National	38.9 ± 9.6	182.5 ± 9.9	108.6 ± 23.3	17.2 ± 8.6	7.4 ± 4.6
Winwood et al. [13]	Survey	73 International	45.1 ± 11.0	183.8 ± 9.4	107.7 ± 20.0	22.7 ± 10.1	12.6 ± 8.3
Powerlifting							
Grgic & Mikulic [12]	Interview	10	29.2 ± 3.2	NS	NS	NS	2.2 ± 1.7^a^
Grgic & Mikulic [12]	Interview	6 ♂ elite	29.8 ± 3.8	NS	86.3 ± 16.8^b^	NS	3.1 ± 1.4^a^
Grgic & Mikulic [12]	Interview	4 ♀ elite	28.3 ± 2.2	NS	64.2 ± 9.4^b^	NS	1.0 ± 1.1^a^
Pritchard et al. [6]	Interview	8 ♂ & 3♀ elite	28.4 ± 7.0	NS	91.0 ± 27.4^b^	NS	NS
Pritchard et al. [6]	Interview	8 ♂ elite	26.5 ± 5.4	NS	94.9 ± 29.9^b^	NS	NS
Pritchard et al. [6]	Interview	3♀ elite	33.3 ± 9.6	NS	80.4 ± 19.4^b^	NS	NS
Travis et al. [11]	Survey	364	AgeCateg	170.4 ± 9.1	WeightCateg	NS	NS
Travis et al. [11]	Survey	225 ♂	AgeCateg	175.0 ± 7.0	WeightCateg	NS	NS
Travis et al. [11]	Survey	139 ♀	AgeCateg	162.9 ± 6.9	WeightCateg	NS	NS
Travis et al. [11]	Survey	103 Local/Reg	AgeCateg	172.0 ± 8.8	WeightCateg	NS	NS
Travis et al. [11]	Survey	200 National	AgeCateg	170.2 ± 9.2	WeightCateg	NS	NS
Travis et al. [11]	Survey	61 International	AgeCateg	168.1 ± 9.1	WeightCateg	NS	NS
Strongman							
Winwood et al. [7]	Survey	454	33.2 ± 8.0	178.1 ± 10.6	108.6 ± 27.9	12.6 ± 8.9	5.3 ± 5.0
Winwood et al. [7]	Survey	353 ♂	33.5 ± 8.3	181.5 ± 8.8	117.1 ± 24.4	14.0 ± 9.0	6.0 ± 5.3
Winwood et al. [7]	Survey	101 ♀	32.1 ± 7.1	165.9 ± 7.0	79.1 ± 17.1	7.6 ± 6.4	2.8 ± 2.8
Winwood et al. [7]	Survey	208 ≤ 30y	26.4 ± 2.9	177.9 ± 9.9	106.2 ± 28.3	7.5 ± 4.3	3.1 ± 3.1
Winwood et al. [7]	Survey	242 ≥ 30y	39.0 ± 6.3	178.3 ± 11.1	110.8 ± 27.4	16.9 ± 9.4	7.1 ± 5.6
Winwood et al. [7]	Survey	224 ≤ 105 kg	31.9 ± 7.5	171.9 ± 8.9	86.1 ± 14.1	10.3 ± 7.8	4.0 ± 4.1
Winwood et al. [7]	Survey	226 ≥ 105 kg	34.2 ± 8.2	184.1 ± 8.4	130.9 ± 18.8	14.7 ± 9.3	6.4 ± 5.2
Winwood et al. [7]	Survey	157 Local/Reg	31.3 ± 7.5	177.3 ± 10.3	103.7 ± 25.6	10.4 ± 8.5	3.3 ± 3.6
Winwood et al. [7]	Survey	212 National Amt	33.7 ± 8.1	177.8 ± 10.7	107.2 ± 27.9	12.5 ± 8.5	5.2 ± 4.6
Winwood et al. [7]	Survey	85 Professional	35.3 ± 8.3	180.4 ± 10.9	121.0 ± 28.7	16.8 ± 9.3	8.8 ± 6.2
Weightlifting							
Winwood et al. [14]	Survey	146	29.2 ± 8.7	172.5 ± 10.1	84.0 ± 17.2	9.5 ± 7.5	4.7 ± 3.4
Winwood et al. [14]	Survey	99 ♂	29.0 ± 7.8	177.9 ± 7.6	88.5 ± 13.4	10.8 ± 8.2	5.0 ± 3.6
Winwood et al. [14]	Survey	47 ♀	28.8 ± 9.3	163.8 ± 6.9	74.3 ± 19.5	6.9 ± 4.6	3.6 ± 1.8
Winwood et al. [14]	Survey	51 Local/Reg	27.9 ± 6.7	176.2 ± 10.0	80.9 ± 18.2	8.2 ± 5.2	3.2 ± 1.6
Winwood et al. [14]	Survey	61 National	27.9 ± 8.4	171.6 ± 10.4	84.6 ± 18.4	8.3 ± 6.3	4.7 ± 2.9
Winwood et al. [14]	Survey	34 International	33.2 ± 9.8	170.8 ± 7.1	80.4 ± 12.0	10.1 ± 5.0	6.7 ± 4.5

a = Calculated from presented athlete’s data, b = Competition body mass, AgeCateg = Age categories presented (e.g. Sub-Jr./junior (18–23y)), *Amt* Amateur, *NS* Not stated, *Reg* Regional level, WeightCateg = Weight categories presented (e.g. LW(M:266 kg;F:252 kg))

### Athletes Training Characteristics

Across the included studies, athletes from the weight lifting sports generally performed between four and five resistance training sessions per week, with training session durations typically ranging from 90 to 120 min (see Table 3). Competition experience varied considerably by athlete level and sport, with international Highland Games heavy event athletes and professional strongman athletes reporting the greatest number of competitive years (12.0 ± 7.5 and 6.5 ± 4.7 y; respectively). Elite CrossFit athletes engaged in the greatest amount of cardiovascular or metabolic training, averaging four sessions weekly, while strongman and Highland Games heavy event athletes completed around two sessions, and weightlifters reported the lowest frequency, with about one session per week. Despite these differences, training session duration was broadly similar across sports, with elite CrossFit, weightlifting and strongman athletes tending toward longer training sessions (≈ 2 h) compared to Highland Games heavy event athletes (83 to 94 min). Male and female athletes followed comparable training structures, although female Highland Games heavy event athletes reported shorter average training durations than their male counterparts (74 ± 25 vs. 94 ± 38 min).

**Table 3:** Summary of the athletes training characteristics reported in the included studies

Study	Athletes	Competition Experience (y)	Weekly Resistance Training Sessions (*n*)	Weekly Cardio/Metabolic Training Sessions (*n*)	Training Duration (min)
CrossFit					
Pritchard et al. [8]	72 elite	4.4 ± 1.7	4.8 ± 2.2	4.1 ± 2.3^ae^ 4.3 ± 2.2^an^	120.7 ± 36.7
Pritchard et al. [8]	39 ♀ elite	4.6 ± 1.7	4.3 ± 1.2	4.1 ± 2.1^ae^ 4.3 ± 2.3^an^	120.1 ± 35.5
Pritchard et al. [8]	33 ♂ elite	4.2 ± 1.6	5.5 ± 2.8	4.2 ± 2.6^ae^ 4.3 ± 2.1^an^	121.5 ± 38.6
Highland Games					
Winwood et al. [13]	169	8.1 ± 6.9	4.7 ± 1.7	2.2 ± 1.6	88 ± 36
Winwood et al. [13]	118 ♂	9.1 ± 7.4	4.9 ± 1.6	1.9 ± 1.6	94 ± 38
Winwood et al. [13]	51 ♀	6.1 ± 4.8	4.1 ± 1.7	2.9 ± 1.5	74 ± 25
Winwood et al. [13]	74 Local/Reg	4.5 ± 4.3	4.9 ± 1.0	2.3 ± 1.7	85 ± 38
Winwood et al. [13]	22 National	7.3 ± 4.7	4.6 ± 1.0	2.1 ± 1.6	83 ± 24
Winwood et al. [13]	73 International	12.0 ± 7.5	4.8 ± 1.3	2.1 ± 1.6	91 ± 36
Powerlifting					
Grgic & Mikulic [12]	10 elite	NS	4.0 ± 1.0	NS	NS
Grgic & Mikulic [12]	6 ♂ elite	NS	NS	NS	NS
Grgic & Mikulic [12]	4 ♀ elite	NS	NS	NS	NS
Pritchard et al. [6]	8 ♂ & 3♀ elite	NS	4.8 ± 0.5	NS	NS
Pritchard et al. [6]	8 ♂ elite	NS	NS	NS	NS
Pritchard et al. [6]	3♀ elite	NS	NS	NS	NS
Travis et al. [11]	364	4.3 ± 3.4	4.4 ± 0.7	NS	1-2 h 222 (61%)^*^ 2-3 h 109 (30%)^*^
Travis et al. [11]	225 ♂	NS	4.4 ± 0.8	NS	1-2 h 126 (56%)^*^ 2-3 h 79 (35%)^*^
Travis et al. [11]	139 ♀	NS	4.3 ± 0.7	NS	1-2 h 96 (69%)^*^ 2-3 h 30 (22%)^*^
Travis et al. [11]	103 Local/Reg	NS	4.4 ± 0.7	NS	1-2 h 68 (66%)^*^ 2-3 h 24 (23%)^*^
Travis et al. [11]	200 National	NS	4.5 ± 0.7	NS	1-2 h 129 (65%)^*^ 2-3 h 58 (29%)^*^
Travis et al. [11]	61 International	NS	4.2 ± 0.8	NS	1-2 h 25 (41%)^*^ 2-3 h 17 (44%)^*^
Strongman					
Winwood et al. [7]	454	4.6 ± 4.4	4.0 ± 1.3	2.2 ± 1.6	111 ± 37
Winwood et al. [7]	353 ♂	5.2 ± 4.7	4.0 ± 1.4	2.1 ± 1.5	112 ± 38
Winwood et al. [7]	101 ♀	2.6 ± 2.6	4.0 ± 1.2	2.4 ± 1.6	106 ± 32
Winwood et al. [7]	208 ≤ 30y	2.6 ± 2.0	3.9 ± 1.1	2.1 ± 1.5	117 ± 36
Winwood et al. [7]	242 ≥ 30y	6.4 ± 5.2	4.0 ± 1.5	2.2 ± 1.6	106 ± 36
Winwood et al. [7]	224 ≤ 105 kg	3.6 ± 3.5	3.9 ± 1.2	2.3 ± 1.6	104 ± 35
Winwood et al. [7]	226 ≥ 105 kg	5.6 ± 4.7	4.0 ± 1.4	2.0 ± 1.4	117 ± 37
Winwood et al. [7]	157 Local/Reg	2.5 ± 2.4	4.0 ± 1.1	2.1 ± 1.5	104 ± 50
Winwood et al. [7]	212 National Amt	4.7 ± 4.0	4.0 ± 1.3	2.3 ± 1.7	110 ± 35
Winwood et al. [7]	85 Professional	8.3 ± 5.6	4.0 ± 1.7	2.0 ± 1.3	127 ± 39
Weightlifting					
Winwood et al. [14]	146	3.9 ± 3.3	4.7 ± 1.2	1.2 ± 1.5	116 ± 33
Winwood et al. [14]	99 ♂	4.3 ± 3.7	4.7 ± 1.0	0.7 ± 1.0	119 ± 29
Winwood et al. [14]	47 ♀	3.0 ± 1.8	4.7 ± 1.1	2.0 ± 1.7	110 ± 34
Winwood et al. [14]	51 Local/Reg	2.3 ± 1.4	4.9 ± 1.0	1.1 ± 1.5	115 ± 29
Winwood et al. [14]	61 National	4.1 ± 2.8	4.6 ± 1.0	1.2 ± 1.4	113 ± 32
Winwood et al. [14]	34 International	6.5 ± 4.7	4.8 ± 1.3	0.9 ± 1.4	125 ± 33

^Ae^ = Aerobic, ^An^ = Anaerobic, Amt = Amateur level, *NS*  Not stated. Reg = Regional level, ^*^ = Highest two categories in hours presented

### Athletes Taper and Coaching Characteristics

Data on athletes’ taper and coaching characteristics across the weight lifting sports are summarized in Table 4. These include the proportion of athletes who implemented a taper, the types and durations of tapers reported, and the extent of coaching support athletes received (coached, partially coached, or self-coached). The following section presents the results for each variable, with subgroup analyses by sex, competitive level, age, and bodyweight reported where applicable.

**Table 4:** Summary of the taper and coaching characteristics reported in the included studies

Study	Athletes	Taper	Taper Type*	Always Taper	Average Taper Length (d)	Coaching*
CrossFit						
Pritchard et al. [8]	72 elite	71 (99%)	Step 28 (42%) Linear 20 (30%)	33 (50%)	5.4 ± 2.7	Coached 54 (75%) Self-Coached 13 (18%)
Pritchard et al. [8]	39 ♀ elite	38 (97%)	Linear 13 (38%) Step 10 (29%)	21 (60%)	NS	Coached 34 (87%) Self-Coached 3 (8%)
Pritchard et al. [8]	33 ♂ elite	33 (100%)	Step 18 (56%) Linear 7 (22%)	12 (39%)	NS	Coached 20 (61%) Self-Coached 10 (30%)
Highland Games						
Winwood et al. [13]	169	132 (78%)	Step 74 (45%) Other 39 (24%)	82 (49%)	5.2 ± 3.5	Self-Coached 106 (63%) Some Coaching 40 (24%)
Winwood et al. [13]	118 ♂	91 (77%)	Step 54 (48%) Other 26 (23%)	56 (49%)	5.3 ± 3.4	Self-Coached 81 (69%) Some Coaching 21 (18%)
Winwood et al. [13]	51 ♀	41 (80%)	Step 20 (40%) Other 13 (26%)	26 (51%)	4.9 ± 3.6	Self-Coached 25 (49%) Some Coaching 19 (37%)
Winwood et al. [13]	74 Local/Reg	56 (76%)	Step 29 (42%) Exp 16 (23%)	41 (57%)	4.7 ± 2.9	Self-Coached 48 (65%) Some Coaching 14 (19%)
Winwood et al. [13]	22 National	18 (82%)	Step 10 (48%) Linear 6 (29%)	9 (43%)	6.4 ± 4.0	Self-Coached 14 (64%) Some Coaching 6 (27%)
Winwood et al. [13]	73 International	58 (80%)	Step 35 (48%) Other 22 (30%)	32 (44%)	5.3 ± 3.8	Self-Coached 44 (60%) Some Coaching 20 (27%)
Powerlifting						
Grgic & Mikulic [12]	10 elite	NS	Exp Fast Decay (60%) Step (40%)	NS	18.0 ± 8.0 (HI 24 ± 4.0, LO 9.0 ± 1.0)	6 Coached 4 Self-Coached
Grgic & Mikulic [12]	6 ♂ elite	NS	NS	NS	NS	NS
Grgic & Mikulic [12]	4 ♀ elite	NS	NS	NS	NS	NS
Pritchard et al. [6]	8 ♂ & 3♀ elite	NS	NS	NS	16.8 ± 6.3^#^	9 Self-Coached 2 Some Coaching
Pritchard et al. [6]	8 ♂ elite	NS	NS	NS	NS	NS
Pritchard et al. [6]	3♀ elite	NS	NS	NS	NS	NS
Travis et al. [11]	364	364 (100%)^»^	Step 164 (45%) Linear 103 (28%)	NS	7–10 days 176 (48%) < 7 days 123 (34%)	Coached 258 (71%) Self-Coached 106 (29%)
Travis et al. [11]	225 ♂	225 (100%)^»^	Step 90 (40%) Linear 59 (26%)	NS	7–10 days 108 (48%) < 7 days 70 (31%)	Coached 136 (60%) Self-Coached 89 (40%)
Travis et al. [11]	139 ♀	139 (100%)^»^	Step 74 (53%) Linear 44 (32%)	NS	7–10 days 68 (49%) < 7 days 53 (38%)	Coached 122 (88%) Self-Coached 17 (12%)
Travis et al. [11]	103 Local/Reg	103 (100%)^»^	Step 47 (46%) Linear 31 (30%)	NS	7–10 days 40 (39%) < 7 days 39 (38%	Coached 63 (61%) Self-Coached 40 (39%)
Travis et al. [11]	200 National	200 (100%)^»^	Step 98 (49%) Linear 51 (26%)	NS	7–10 days 112 (56%) < 7 days 59 (30%)	Coached 152 (76%) Self-Coached 48 (24%)
Travis et al. [11]	61 International	61 (100%)^»^	Linear 19 (34%) Step 21 (31%)	NS	< 7 days 25 (41%) 7–10 days 24 (39%)	Coached 43 (70%) Self-Coached 18 (30%)
Strongman						
Winwood et al. [7]	454	396 (87%)	Step 131 (52%) Linear 42 (17%)	198 (80%)	8.6 ± 5.0	Self-Coached 239 (53%) Coached 171 (38%)
Winwood et al. [7]	353 ♂	300 (85%)	Step 105 (54%) Exp 31 (16%)	148 (77%)	8.3 ± 4.8	Self-Coached 217 (62%) Coached 101 (29%)
Winwood et al. [7]	101 ♀	95 (94%)	Step 26 (46%) Linear 12 (21%)	50 (89%)	9.5 ± 5.5	Coached 75 (74%) Self-Coached 22 (22%)
Winwood et al. [7]	208 ≤ 30y	184 (88%)	Step 60 (58%) Linear 15 (14%)	87 (84%)	8.2 ± 4.2	Self-Coached 96 (46%) Coached 91 (44%)
Winwood et al. [7]	242 ≥ 30y	208 (86%)	Step 71 (49%) Linear 27 (19%)	110 (77%)	9.0 ± 5.7	Self-Coached 141 (59%) Coached 83 (34%)
Winwood et al. [7]	224 ≤ 105 kg	200 (90%)	Step 75 (59%) Linear 21 (16%)	107 (84%)	8.6 ± 4.9	Coached 111 (50%) Self-Coached 90 (40%)
Winwood et al. [7]	226 ≥ 105 kg	193 (85%)	Step 56 (46%) Linear 21 (17%)	90 (76%)	8.7 ± 5.2	Self-Coached 146 (65%) Coached 64 (28%)
Winwood et al. [7]	157 Local/Reg	139 (89%)	Step 50 (57%) Linear 18 (20%)	79 (90%)	8.6 ± 5.0	Self-Coached 75 (48%) Coached 69 (44%)
Winwood et al. [7]	212 National Amt	185 (87%)	Step 61 (54%) Exp 20 (18%)	85 (75%)	9.0 ± 5.4	Self-Coached 107 (51%) Coached 88 (42%)
Winwood et al. [7]	85 Professional	71 (84%)	Step 20 (42%) Other 10 (21%)	34 (74%)	7.8 ± 4.1	Self-Coached 57 (67%) Coached 19 (22%)
Weightlifting						
Winwood et al. [14]	146	144 (99%)	Linear 31 (36%) Step 28 (33%)	42 (49%)	8.0 ± 4.4	Coached 106 (73%) Self-Coached 34 (23%)
Winwood et al. [14]	99 ♂	97 (98%)	Step 24 (35%) Linear 23 (33%)	31 (45%)	8.6 ± 4.6	Coached 62 (63%) Self-Coached 32 (32%)
Winwood et al. [14]	47 ♀	47 (100%)	Linear 8 (47%) Step 4 (24%)	11 (65%)	5.3 ± 1.8	Coached 44 (94%) Self-Coached 2 (4%)
Winwood et al. [14]	51 Local/Reg	50 (98%)	Step 15 (48%) Linear 9 (29%)	12 (39%)	8.2 ± 4.0	Coached 31 (61%) Self-Coached 19 (37%)
Winwood et al. [14]	61 National	60 (98%)	Linear 14 (39%) Exp 13 (36%)	21 (58%)	7.9 ± 4.2	Coached 48 (79%) Self-Coached 10 (16%)
Winwood et al. [14]	34 International	34 (100%)	Linear 8 (42%) Step 5 (26%)	9 (47%)	7.8 ± 5.5	Coached 27 (79%) Self-Coached 5 (15%)

*AMT* Amateur, *Exp* Exponential, *HI* Wilks coefficient score > 350, LO Wilks coefficient score < 350, NS Not stated, Reg Regional level, ^#^ = Calculated from reported weeks value,^»^ = Study included only powerlifters who utilized a taper, * = Highest two categories presented

#### Taper Prevalence

Across the included studies, the majority of athletes reported implementing a taper prior to competition. In powerlifting, the study by Travis and colleagues [13] only included athletes who reported tapering, resulting in a prevalence of 100%. Similarly high rates were observed in CrossFit (99%) and weightlifting (99%). Slightly lower proportions were reported among strongman (87%) and Highland Games heavy event athletes (78%). Subgroup analyses showed that, in all cases except powerlifting (which was uniformly 100%), female athletes reported higher tapering prevalence than male athletes. International-level athletes across all sports, with the exception of strongman, were also more likely to taper than their local or regional counterparts.

The proportion of athletes who reported always tapering prior to competition varied across sports. Strongman athletes demonstrated the highest prevalence, with 80% consistently tapering. In contrast, approximately half of athletes reported always tapering in CrossFit (50%), Highland Games (49%), and weightlifting (49%). Data on this outcome were not reported in the powerlifting studies. By sex, females generally reported higher rates of always tapering compared to males. This pattern was evident in CrossFit (60% vs. 39%), weightlifting (65% vs. 45%), and strongman (89% vs. 77%). In the Highland Games, rates were similar between sexes (51% vs. 49%). Subgroup analyses showed variation in the proportion of athletes who always tapered across sports. In the Highland Games, prevalence was higher in local/regional athletes (57%) compared with national and international (43% and 44%) competitors. Among strongman athletes, younger (≤ 30 y, 84%) and lighter (≤ 105 kg, 84%) athletes were more likely to report always tapering than older (≥ 30 y, 77%) and heavier (≥ 105 kg, 76%) athletes; rates were also highest among local/regional competitors (90%) compared with national amateurs and professionals (75% and 74%). In weightlifting, fewer athletes reported always tapering, with local/regional lifters showing the lowest prevalence (39%) compared with national and international athletes (58% and 47%, respectively).

#### Taper Type

The type of taper adopted varied across the weight lifting sports. In CrossFit, step tapers were most commonly reported (42%), followed by linear tapers (30%). Highland Games heavy event athletes also most frequently used a step taper (45%) with “other” taper formats (e.g. own type of taper or training cessation) the next most prevalent (24%). In powerlifting, taper type differed across studies: Grgic and Mikulic [14] reported exponential fast-decay tapers as most common (60%), followed by step tapers (40%), whereas Travis and colleagues [13] observed step (45%) and linear (28%) tapers as the predominant strategies. Among strongman athletes, step tapering was most frequently reported (52%), with linear tapering also used (17%). Weightlifters demonstrated a more even distribution, with linear (36%) and step (33%) tapers the most common. By sex, males more frequently reported step tapering, whereas females demonstrated a greater reliance on linear formats in some sports. In CrossFit and weightlifting, females favored linear tapers (38% and 47%, respectively) compared with males, who predominantly used step tapers (56% and 35%). In Highland Games and strongman, both sexes preferred step tapers, although males reported higher proportions than females. In powerlifting, females again showed higher usage of both step (53%) and linear (32%) tapers compared with males (40% and 26%).

Subgroup analyses of taper type highlighted some variation across competition level, age, and bodyweight categories. In the Highland Games, step tapers were the most frequently reported across all levels, with a greater proportion observed at national and international (both 48%) levels, while other taper formats were more commonly reported among international athletes (30%). In powerlifting, step tapering predominated at local/regional (46%) and national (49%) levels, whereas international lifters reported a more even distribution, with linear (34%) and step (31%) tapers most common. Strongman athletes consistently favored step tapering across subgroups, though proportions were higher in younger (≤ 30 y, 58%) and lighter (≤ 105 kg, 59%) athletes compared to their older (≥ 30 y, 49%) and heavier (≥ 105 kg, 46%) counterparts. At the competition level, step tapering was more prevalent in local/regional and national amateur strongman, while professionals reported a more varied use of taper formats, including step (42%) and other approaches (21%). Weightlifting athletes showed variation by competition level, with local/regional athletes most often using step tapers (48%), national athletes reporting comparable proportions of linear (39%) and exponential (36%) tapers, and international athletes favoring linear formats (42%).

#### Taper Duration

The reported duration of taper varied across sports. Elite CrossFit and Highland Games heavy event athletes tapered for similar lengths of time, averaging 5.4 ± 2.7 and 5.2 ± 3.5 days, respectively. Strongman and weightlifters reported slightly longer taper periods of 8.6 ± 5.0 and 8.0 ± 4.4 days. In powerlifting, taper duration was more variable across studies, with mean values of 18.0 ± 8.0 days and 16.8 ± 6.3 days reported in two studies [4, 14], while categorical data indicated that 48% of athletes tapered for 7–10 days and 34% for fewer than 7 days [13]. Subgroup analyses indicated some variation in taper length by sex across sports. In both the Highland Games and powerlifting [13], taper duration patterns were broadly similar between males and females. Among strongman athletes, females reported slightly longer tapers (9.5 ± 5.5 days) compared with males (8.3 ± 4.8 days). In weightlifting, taper duration differed more notably, with males averaging 8.6 ± 4.6 days and females 5.3 ± 1.8 days. When examined by competition level, age, and bodyweight, taper lengths were generally consistent across sports. In the Highland Games, durations were broadly similar across levels (≈5–6 days), while the powerlifting survey data [13] showed a distinct pattern, with national athletes more often tapering for 7–10 days and international lifters tending toward shorter tapers (< 7 days). Strongman athletes and weightlifters demonstrated little variation by subgroup, averaging around 8–9 days.

#### Coaching Characteristics

The prevalence of coaching support varied across sports. In CrossFit and weightlifting, the majority of athletes reported being coached (75% and 73%, respectively). In contrast, strongman and Highland Games heavy event athletes were more self-coached (63% and 73%, respectively). Powerlifting showed mixed patterns across studies, with one reporting nearly equal numbers of coached and self-coached athletes [14], another indicating a predominance of self-coached lifters [4], and the other demonstrating that most athletes were coached (71%) [13]. Subgroup analyses by sex revealed differences in access to coaching across sports. In CrossFit and powerlifting, females were more likely to be coached (87% and 88%) compared with males (61% and 62%). A similar pattern was observed in strongman, where most females were coached (74%), while the majority of males were self-coached (62%). Among Highland Games heavy event athletes, self-coaching was common in both sexes, though more prevalent in males (69% vs. 49%), whereas females were more likely to report some level of coaching support (37% vs. 18%).

Across subgroups, coaching support was most consistently reported in powerlifting and weightlifting, where athletes at higher competitive levels were more likely to work with formal coaches. Coaching prevalence increased with competitive level in both sports, with 76% of national and 70% of international lifters in powerlifting [13], and 79% of weightlifters (at both levels), reporting access to coaching. In contrast, Highland Games heavy event athletes were most often self-coached across levels (range 60% to 65%), with only 19–27% reporting some coaching. Strongman athletes showed more variation: younger athletes (≤ 30 y) reported similar proportions of coaching (44%) and self-coaching (46%), while older athletes (≥ 30 y) were more frequently self-coached (59%). By body mass, lighter strongman athletes (≤ 105 kg) reported more coaching (50%) than heavier athletes (≥ 105 kg), among whom 65% were self-coached. At the professional level, two-thirds of strongman athletes (67%) were self-coached compared with 48% and 51% at local/regional and national amateur level (respectively).

### Athletes Taper Training Characteristics

Data on athletes taper training characteristics across the weight lifting sports are summarized in Table 5. These include measures such as training cessation before competition, timing of highest training volume and intensity, reductions in training volume during the taper, and the timing of the final training at loads > 85% 1RM. The following section presents the results for each variable, with subgroup analyses by sex, competitive level, age, and bodyweight reported where applicable.

**Table 5:** Summary of the taper training characteristics reported in the included studies

Study	Athletes	Cease training before comp (d)	Weeks out highest training volume (wk)*	Weeks out highest training intensity (wk)	Drop in training volume during taper (%)	Final training session at loads > 85% 1RM (d)ǂ
CrossFit						
Pritchard et al. [8]	72 elite	2.0 ± 1.1	CT 4.0 ± 4.4 ST 5.1 ± 4.6	CT 2.8 ± 2.2 ST 3.1 ± 2.4	41 ± 16	5.8 ± 3.2
Pritchard et al. [8]	39 ♀ elite	NS	NS	NS	42 ± 13	NS
Pritchard et al. [8]	33 ♂ elite	NS	NS	NS	40 ± 18	NS
Highland Games						
Winwood et al. [13]	169	2.4 ± 1.4	5.5 ± 5.7	3.8 ± 3.3	34.1 ± 24.9	NS
Winwood et al. [13]	118 ♂	2.6 ± 1.5	5.7 ± 5.7	3.7 ± 3.3	34.3 ± 24.2	NS
Winwood et al. [13]	51 ♀	1.9 ± 1.1	3.8 ± 3.4	3.9 ± 3.5	33.5 ± 26.7	NS
Winwood et al. [13]	74 Local/Reg	2.3 ± 1.6	5.7 ± 3.2	3.9 ± 3.3	35.2 ± 26.7	NS
Winwood et al. [13]	22 National	2.4 ± 1.2	4.6 ± 2.6	3.9 ± 3.0	23.8 ± 22.8	NS
Winwood et al. [13]	73 International	2.5 ± 1.4	5.4 ± 5.8	3.7 ± 3.5	35.3 ± 23.8	NS
Powerlifting						
Grgic & Mikulic [12]	10 elite	3.0 ± 1.0	4.5 ± 1.8	1.1 ± 0.4^#^	50.5 ± 11.7	SQ 7.0 ± 1.0 BP 6.0 ± 2.0 DL 8.0 ± 2.0
Grgic & Mikulic [12]	6 ♂ elite	NS	NS	NS	NS	NS
Grgic & Mikulic [12]	4 ♀ elite	NS	NS	NS	NS	NS
Pritchard et al. [6]	8 ♂ & 3♀ elite	3.7 ± 1.6	5.2 ± 1.7	1.9 ± 0.8	58.9 ± 8.4	SQ 8.0 ± 2.9 BP 7.3 ± 2.7 DL 10.9 ± 4.0
Pritchard et al. [6]	8 ♂ elite	NS	NS	NS	NS	NS
Pritchard et al. [6]	3♀ elite	NS	NS	NS	NS	NS
Travis et al. [11]	364	2.8 ± 1.1	5–8 weeks (45%) ≤ 4 weeks (30%)	2.3 ± 1.2	41 to 50^«^	SQ 7–10 days (48%) BP < 7 days (63%) DL 7–10 days (55%)
Travis et al. [11]	225 ♂	2.9 ± 1.0	5–8 weeks (48%) ≤ 4 weeks (29%)	2.3 ± 1.2	NS	SQ 7–10 days (47%) BP < 7 days (62%) DL 7–10 days (51%)
Travis et al. [11]	139 ♀	2.7 ± 1.1	5–8 weeks (39%) ≤ 4 weeks (32%)	2.3 ± 1.2	NS	SQ 7–10 days (50%) BP < 7 days (66%) DL 7–10 days (63%)
Travis et al. [11]	103 Local/Reg	2.9 ± 1.2	5–8 weeks (46%) ≤ 4 weeks (32%)	2.2 ± 1.1	NS	SQ 7–10 days (44%) BP < 7 days (60%) DL 7–10 days (53%)
Travis et al. [11]	200 National	2.8 ± 1.0	5–8 weeks (47%) ≤ 4 weeks (28%)	2.3 ± 1.1	NS	SQ 7–10 days (51%) BP < 7 days (69%) DL 7–10 days (56%)
Travis et al. [11]	61 International	3.0 ± 1.1	5–8 weeks (34%) ≤ 4 weeks (34%)	2.4 ± 1.0	NS	SQ 7–10 days (48%) BP < 7 days (51%) DL 7–10 days (57%)
Strongman						
Winwood et al. [7]	454	3.9 ± 1.8	4.3 ± 2.9	2.5 ± 0.9	45.5 ± 12.9	9.1 ± 3.3
Winwood et al. [7]	353 ♂	4.0 ± 1.8	4.5 ± 3.0	2.5 ± 0.9	45.1 ± 12.3	9.2 ± 3.3
Winwood et al. [7]	101 ♀	3.8 ± 1.7	3.9 ± 2.5	2.5 ± 0.9	46.7 ± 14.8	8.6 ± 3.0
Winwood et al. [7]	208 ≤ 30y	3.7 ± 1.6	4.6 ± 3.1	2.4 ± 0.9	45.8 ± 12.9	9.1 ± 3.3
Winwood et al. [7]	242 ≥ 30y	4.1 ± 1.9	4.1 ± 2.7	2.6 ± 0.9	45.1 ± 13.1	9.1 ± 3.3
Winwood et al. [7]	224 ≤ 105 kg	4.0 ± 1.8	4.3 ± 2.9	2.5 ± 0.9	45.6 ± 13.3	9.1 ± 3.2
Winwood et al. [7]	226 ≥ 105 kg	3.9 ± 1.8	4.4 ± 2.9	2.5 ± 0.9	45.4 ± 12.6	9.1 ± 3.2
Winwood et al. [7]	157 Local/Reg	3.8 ± 1.8	4.2 ± 2.8	2.5 ± 0.8	44.8 ± 13.2	9.3 ± 3.3
Winwood et al. [7]	212 NationalAmt	3.9 ± 1.7	4.2 ± 2.9	2.5 ± 1.0	45.6 ± 13.1	9.0 ± 3.3
Winwood et al. [7]	85 Professional	4.3 ± 2.0	5.0 ± 2.7	2.6 ± 0.9	46.5 ± 12.0	8.7 ± 3.1
Weightlifting						
Winwood et al. [14]	146	1.5 ± 0.6	5.9 ± 3.9	2.6 ± 1.5	43.1 ± 14.6	5.9 ± 2.3
Winwood et al. [14]	99 ♂	1.5 ± 0.6	6.0 ± 3.7	2.6 ± 1.4	43.6 ± 14.4	5.9 ± 2.2
Winwood et al. [14]	47 ♀	1.5 ± 0.6	5.4 ± 4.6	2.5 ± 1.7	40.7 ± 15.8	5.8 ± 2.8
Winwood et al. [14]	51 Local/Reg	1.4 ± 0.6	5.7 ± 3.2	2.3 ± 1.1	45.8 ± 13.9	5.4 ± 1.8
Winwood et al. [14]	61 National	1.5 ± 0.6	5.8 ± 4.1	2.8 ± 1.7	39.4 ± 11.9	6.1 ± 2.6
Winwood et al. [14]	34 International	1.5 ± 0.7	6.5 ± 4.6	2.7 ± 1.5	45.6 ± 19.5	6.1 ± 2.6

*AMT* Amateur, *BP* Bench Press, *CT* Conditioning Training, *DL* Deadlift, *NS* Not stated, Reg Regional level, *SQ* Squat, *ST* Strength Training,* = Highest two categories presented when data not reported as mean ± SD, ǂ = Highest category presented when data not reported as mean ± SD, ^#^ calculated from days reported. ^«^Most common range reported by 94% of athletes

#### Training Cessation

The number of days athletes ceased training before competition varied across sports. Weightlifters reported the shortest cessation period (1.5 ± 0.6 days), followed by CrossFit (2.0 ± 1.1 days) and Highland Games heavy event athletes (2.4 ± 1.4 days). Powerlifting data ranged across studies from 2.8 ± 1.1 to 3.7 ± 1.6 days, while strongman athletes reported the longest cessation (3.9 ± 1.8 days). Subgroup analysis revealed that training cessation before competition differed by sex among strongman and Highland Games heavy event athletes, with males reporting longer rest periods (4.5 ± 3.0 and 2.6 ± 1.5 days, respectively) compared to females (3.9 ± 2.5 and 1.9 ± 1.1 days, respectively). In contrast, powerlifting, strongman and weightlifting showed little variation in cessation duration across sex or competitive level, though it should be noted that only one powerlifting study [13] provided stratified data.

#### Highest Training Volume

The weeks out of highest training volume before competition was broadly consistent across weight lifting sports, typically occurring between four and six weeks out. The powerlifting study of Travis and colleagues [13] reported categorical ranges; 5–8 weeks before competition (45%) and < 4 weeks (30%). Subgroup analysis revealed notable differences by sex and competitive level. Across multiple sports, females tended to maintain higher training volumes closer to competition than males, as seen in weightlifting (5.4 vs. 6.0 weeks), strongman (3.8 vs. 4.0 weeks), and Highland Games (3.8 vs. 5.7 weeks). Professional and international athletes generally reported reaching their highest training volumes further from competition than local or national athletes, with international weightlifters (6.5 vs. 5.7–5.8 weeks), professional strongman (4.3 vs. 3.7–3.8 weeks), and international Highland Games heavy event athletes (5.4 vs. 4.6 weeks) showing this trend.

#### Highest Training Intensity

The point at which athletes reached their highest training intensity before competition was broadly similar across weight lifting sports, typically occurring between two and three weeks out. CrossFit athletes reported reaching their highest training intensity 2.8 weeks before competition for cardiovascular training and 3.1 weeks for strength training, while Highland Games heavy event athletes reached their highest intensity at 3.8 ± 3.3 weeks. Strongman and weightlifting athletes followed a similar pattern, with peaks at 2.5 ± 0.9 and 2.6 ± 1.5 weeks, respectively. Powerlifting data were more variable across studies, ranging from 1.1 ± 0.4 to 2.3 ± 1.2 weeks. Subgroup analysis showed consistent results across sports regarding the timing of highest training intensity. In weightlifting, however, some variation was observed, with national and international lifters reporting their highest intensity slightly further out (2.8 and 2.7 weeks, respectively) compared to local/regional lifters (2.3 weeks).

#### Drop in Training Volume

Reductions in training volume during the taper among the weight lifting sports ranged from 34 to 59%. Similar values were reported among CrossFit (41.0%), weightlifting (43.1%) and strongman athletes (45.5%), whereas Highland Games heavy event athletes reported the smallest training volume reductions (34.1%). In powerlifting, findings were more variable, with Grgic et al. [14] reporting a 50.5% reduction, Pritchard et al. [4] 58.9%, and Travis et al. [13] between 41 and 50%. Subgroup analysis across sports showed that reductions in training volume during the taper were broadly consistent between sexes, age groups, and weight classes, with only small differences observed. Differences were observed among Highland Games heavy event athletes, with national level athletes reporting smaller reductions (23.8%) compared to local/regional and international level competitors (both 35%). Similarly, in weightlifting larger reductions were observed among national lifters (39.4%) than local/regional and international level athletes (both 45%).

#### Final Training Session (at Loads > 85% 1RM)

The timing of the last heavy training session (> 85% 1RM) before competition varied across sports. In CrossFit and weightlifting, the final heavy session occurred 5.8–5.9 days prior to competition. Strongman athletes reported longer intervals, completing their final heavy session 9.1 ± 3.3 days before competition. In powerlifting, data were categorized by the three competition lifts rather than reported as a single mean value. Grgic et al. [14] reported the final squat, bench press, and deadlift sessions at 7.0 ± 1.0, 6.0 ± 2.0, and 8.0 ± 2.0 days out, respectively. Pritchard et al. [4] reported slightly longer durations, with squats at 8.0 ± 2.9, bench press at 7.3 ± 2.7, and deadlifts at 10.9 ± 4.0 days. Travis et al. [13] reported categorical ranges, with 48% of lifters performing their final squat 7–10 days out, 63% performing their final bench press within < 7 days, and 55% completing their final deadlift 7–10 days out.

Subgroup data were limited, but some consistent patterns were evident. Across powerlifting studies, the deadlift was typically performed furthest out from competition, followed by the squat, with the bench press performed closest to competition. These patterns were consistent across sex and competitive level in the survey data [13]. In weightlifting, small differences were observed by competitive level, with local lifters reporting 5.4 days, national lifters international lifters 6.0 and 6.1 days (respectively). Strongman athletes showed minimal subgroup variation, with final heavy sessions typically around 9 days regardless of age, weight, or level. CrossFit data were not stratified but showed a consistent pattern of ~ 6 days out.

### Main Exercises and Sport-Specific Events When Last Performed and Loads Used (%1RM)

Table 6 summarizes the main exercises and sport-specific events reported in the included studies, detailing when they were last performed during the taper and the relative loads used (%1RM). Data are presented across common strength exercises (squat, bench press, and deadlift), weightlifting movements, overhead and assistance exercises, as well as sport-specific events from the Highland Games and strongman competitions. The following results describe the timing and intensity of these final sessions, with subgroup differences highlighted where available.

**Table 6:** Main exercises and sport-specific events from included studies, including when last performed during the taper and loads used (%1RM)

Sport	Study	Exercise	When Last Performed (d)	Load (%1RM)
		Squat Movements		
CrossFit	Pritchard et al. [8]	Back Squat	3.8 ± 1.6	73.6 ± 10.4
Highland Games	Winwood et al. [13]	Squat	4.8 ± 1.8	68.0 ± 17.6
Powerlifting	Grgic and Mikulic [12]	Squat	4.0 ± 2.0	65.9 ± 20.3
	Pritchard et al. [6]	Squat	4.0 ± 1.8	66.0 ± 15.7
	Travis et al. [11]	Squat	4.1 ± 1.9	NS
Strongman	Winwood et al. [7]	Squat	6.6 ± 3.2	72.6 ± 15.4
Weightlifting	Winwood et al. [14]	Back Squat	5.2 ± 3.2	82.4 ± 10.1
		Front Squat	4.0 ± 2.1	79.2 ± 10.8
		Deadlift		
CrossFit	Pritchard et al. [8]	Deadlift	5.3 ± 2.9	70.0 ± 13.7
Highland Games	Winwood et al. [13]	Deadlift	5.7 ± 2.9	70.1 ± 17.6
Powerlifting	Grgic and Mikulic [12]	Deadlift	4.0 ± 3.0	65.8 ± 19.7
	Pritchard et al. [6]	Deadlift	7.4 ± 4.1	72.6 ± 18.5
	Travis et al. [11]	Deadlift	5.8 ± 2.5	NS
Strongman	Winwood et al. [7]	Deadlift	7.8 ± 3.2	77.0 ± 15.5
Weightlifting	Winwood et al. [14]	Deadlift	7.8 ± 5.0	84.8 ± 11.4
		Bench Press		
Highland Games	Winwood et al. [13]	Bench Press	4.2 ± 1.6	69.1 ± 17.8
Powerlifting	Grgic and Mikulic [12]	Bench Press	3.0 ± 1.0	65.2 ± 20.3
	Pritchard et al. [6]	Bench Press	4.0 ± 1.8	67.3 ± 18.1
	Travis et al. [11]	Bench Press	3.9 ± 1.8	NS
Strongman	Winwood et al. [7]	Bench Press	5.7 ± 2.1	74.1 ± 15.8
		Weightlifting Movements		
CrossFit	Pritchard et al. [8]	Clean & Jerk	3.6 ± 1.3	71.4 ± 10.3
		Snatch	2.6 ± 1.2	74.7 ± 12.1
Highland Games	Winwood et al. [13]	Olympic Lifts	3.2 ± 1.3	64.3 ± 16.6
Weightlifting	Winwood et al. [14]	Clean	3.6 ± 2.6	75.3 ± 15.0
		Snatch	3.1 ± 1.9	74.9 ± 15.1
		Power Snatch	2.5 ± 1.0	70.3 ± 11.1
		Power Clean	2.8 ± 1.1	70.1 ± 10.1
		Jerk	2.6 ± 1.2	74.3 ± 11.6
		Overhead Press		
Highland Games	Winwood et al. [13]	Overhead Press	3.9 ± 1.6	64.8 ± 17.9
Strongman	Winwood et al. [7]	Overhead Press	5.3 ± 1.9	71.5 ± 16.3
Weightlifting	Winwood et al. [14]	Overhead Press	5.7 ± 4.3	73.2 ± 10.9
		Assistance Exercises		
Highland Games	Winwood et al. [13]	Assistance Exercises	3.5 ± 2.0	54.8 ± 9.9
Weightlifting	Winwood et al. [14]	Assistance Exercises	5.7 ± 4.1	65.0 ± 15.8
		Highland Games Events		
Highland Games	Winwood et al. [13]	Heavy Weight Throw	4.9 ± 2.4	NS
		Stone Put	3.1 ± 1.6	NS
		Hammer Throw	3.8 ± 1.5	NS
		Light Weight Throw	3.1 ± 1.5	NS
		Weight for Height	4.7 ± 2.0	NS
		Caber Toss	6.7 ± 3.7	NS
		Sheaf Toss	3.1 ± 1.8	NS
		Strongman Events		
Strongman	Winwood et al. [7]	Log Lift/Press	5.7 ± 2.0	77.0 ± 15.9
		Yoke Walk	8.2 ± 3.5	79.0 ± 16.2
		Farmers Walk	6.1 ± 1.8	77.1 ± 16.5
		Stone Lifts/Work	7.6 ± 2.8	80.4 ± 13.7

*NS* not stated

#### Squat

Across sports, the last squat session was typically performed between 3.8 and 6.6 days before competition, with moderate-to-heavy loads (66–82% 1RM). In CrossFit, the back squat was last performed 3.8 ± 1.6 days out at 73.6 ± 10.4% 1RM. Highland Games athletes reported similar timing, with the final squat completed 4.8 ± 1.8 days out at 68.0 ± 17.6% 1RM. In powerlifting, findings were consistent across studies, with squats performed approximately four days before competition with moderate loads (66% 1RM). Strongman athletes completed their final squat further from competition at 6.6 ± 3.2 days, with higher relative loads (72.6 ± 15.4% 1RM). Weightlifting data indicated higher intensities, with back squats performed 5.2 ± 3.2 days out at 82.4 ± 10.1% 1RM and front squats 4.0 ± 2.1 days out at 79.2 ± 10.8% 1RM.

#### Deadlift

The timing of the last deadlift session before competition ranged from 4.0 to 7.8 days out, with relative loads between ~ 66% and 85% 1RM. In CrossFit and Highland Games, the final deadlift was performed 5.3 and 5.7 days (respectively) before competition at 70% 1RM. Powerlifting findings varied, with the final deadlift performed between 4.0 and 7.4 days before competition, at relative loads ranging from 66 to 73% 1RM. Strongman and weightlifting athletes both reported their final deadlift sessions 7.8 days before competition. However, the relative loads used differed between the two sports, with strongman athletes training at 77.0 ± 15.5% 1RM, while weightlifters employed the heaviest intensities across all sports at 84.8 ± 11.4% 1RM.

#### Bench Press

The timing of the final bench press session before competition was generally shorter than for squats or deadlifts, with most sports performing this exercise within 3–6 days of competition. In the Highland Games, athletes reported completing their last bench press 4.2 ± 1.6 days out at 69.1 ± 17.8% 1RM. In powerlifting, values were consistent across studies, ranging from 3.0 to 4.0 days, at relative loads between ~ 65% and 67% 1RM. Strongman athletes allowed slightly longer intervals, performing their final bench press 5.7 ± 2.1 days out, at heavier relative loads of 74.1 ± 15.8% 1RM.

#### Weightlifting Movements

The final performance of weightlifting movements across sports were performed closer to competition than the squat, deadlift and bench press lifts (within 2–4 days). Loads were moderate to heavy, typically between ~ 70–75% 1RM, though Highland Games heavy event athletes used slightly lighter intensities (64%). In weightlifting, a consistent pattern emerged: technical lifts such as the snatch, clean, and jerk were scheduled within 2.6 to 3.6 days of competition, while power variations (e.g., power snatch, power clean) tended to be performed slightly earlier (2.5 to 2.8), often at similar or slightly lower loads (both at ~ 70% 1RM).

#### Overhead Press

The final overhead press session was generally completed 4–6 days before competition, with moderate to heavy relative loads (64.8 to 73.2%1RM). Highland Games heavy event athletes performed the lift closest to competition (~ 4 days out) and with lighter loads (~ 65% 1RM). Strongman and weightlifting athletes reported longer lead-ins (~ 5–6 days) than Highland Games heavy event athletes (3.9 days), with heavier relative intensities (≈71–73% 1RM).

#### Assistance Exercises

Data on assistance exercises were reported in two studies. Highland Games heavy event athletes scheduled assistance exercises closest to competition (~ 3.5 days out) at ~ 55% 1RM, whereas weightlifters allowed a longer lead-in (~ 5.7 days) and used heavier intensities (~ 65% 1RM).

#### Highland Games Events

Sport-specific Highland Games heavy events were generally performed within the final week before competition, though timing varied by discipline. Most events, such as the stone put, light weight throw, and sheaf toss, were performed closest to competition (≈3 days out), while the hammer throw and weight for height were scheduled slightly later (≈4–5 days). The heavy weight throw was typically performed 4.9 ± 2.4 days out, and the caber toss was performed furthest from competition, at 6.7 ± 3.7 days.

#### Strongman Events

Strongman athletes performed their sport-specific events within the final week before competition, though timing varied by discipline. The log press and farmers walk were completed closer to competition (5.7 and 6.1 days, respectively), while stone lifting and the yoke walk were performed further out (7.6 and 8.2 days, respectively). Loads across all events were consistently heavy, averaging between 77 and 80% 1RM.

### Training Intensity, Frequency, and Duration Taper Changes

**Fig. 2:**
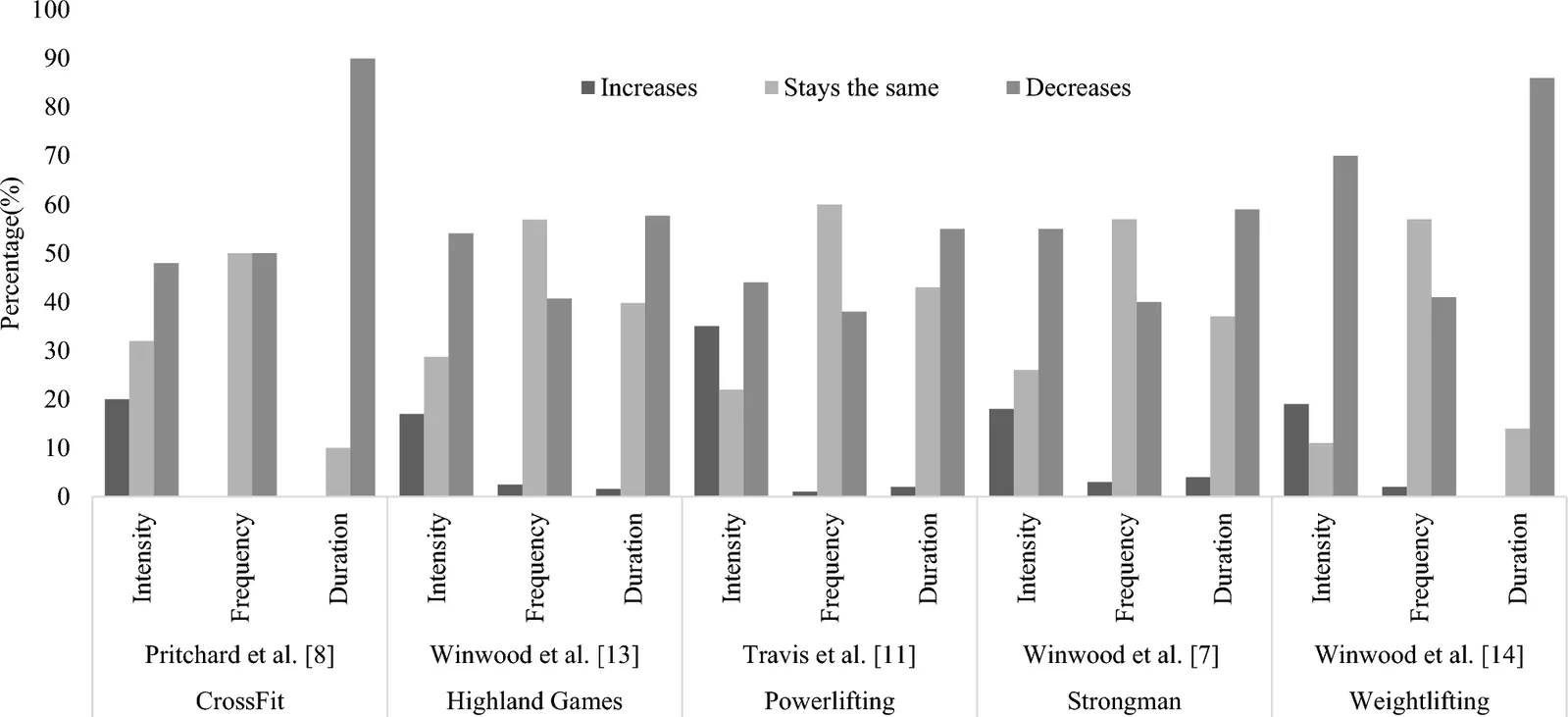
Summary of reported data from studies showing how training intensity, frequency, and duration change during the taper

The summary from studies showing how training intensity, frequency, and duration change during the taper is presented in Fig. 2. Across sports, the most consistent change during the taper was a reduction in training duration, reported by the majority of athletes (55–90%). CrossFit and weightlifting athletes reported the sharpest reductions, while strongman and Highland Games heavy event athletes showed more moderate reductions and placed greater emphasis on maintaining training frequency. Training intensity showed more variability: most athletes in weightlifting (↓70%), strongman (↓55%), Highland Games (↓54%), and CrossFit (↓48%) decreased intensity, while one powerlifting study (Travis et al.) [13] reported an increase (↑35%). Training frequency was largely maintained in all sports (50–60%), with decreases observed in 38–50% of studies.

### Training Types Performed During the Taper

**Fig. 3:**
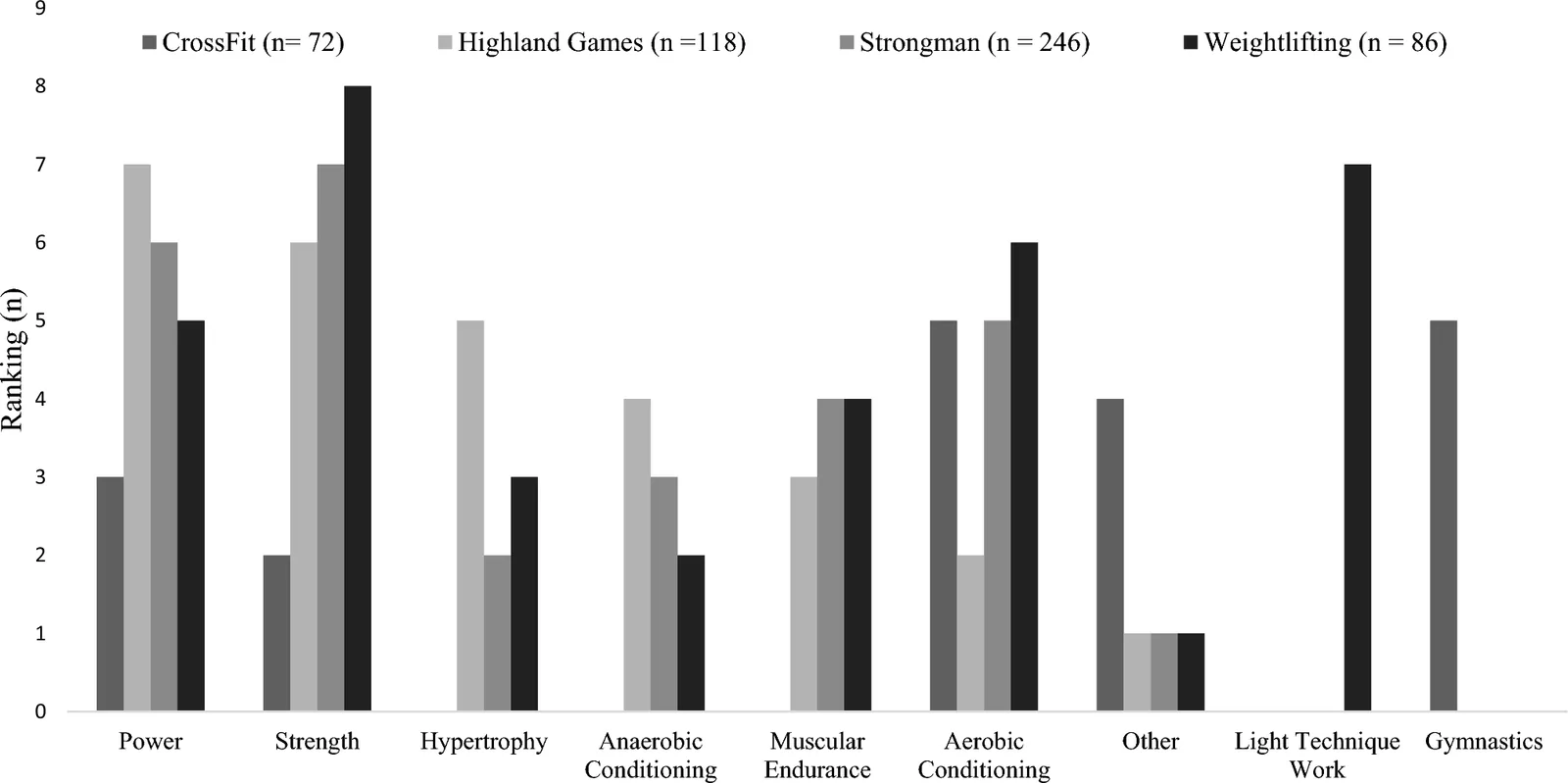
Training types performed by CrossFit, Highland Games, strongman and weightlifting athletes during the taper ranked from highest (most commonly performed) to lowest. Note: Ranking scores were derived from weighted values, calculated according to the number of training types reported. For example, if five training variables were reported, the highest possible rank was 5. For CrossFit, training types were originally reported as monostructural, weightlifting, and powerlifting. These were reclassified as aerobic training, power training, and strength training, respectively

The relative emphasis placed on different training types during the taper varied across sports (see Fig. 3). CrossFit athletes prioritized aerobic conditioning and gymnastics, while also incorporating other training modalities. Highland Games heavy event athletes placed greatest emphasis on power, strength, and hypertrophy, followed by anaerobic conditioning and muscular endurance. Strongman athletes also prioritized power and strength, but with a more evenly distributed focus across aerobic and anaerobic conditioning and moderate attention to muscular endurance. In contrast, weightlifters reported the strongest emphasis on strength and power alongside a distinct focus on technical work (snatch, clean, and jerk), while placing the least priority on anaerobic conditioning and hypertrophy training.

#### Other Types of Strategies Utilized in the Taper

**Fig. 4:**
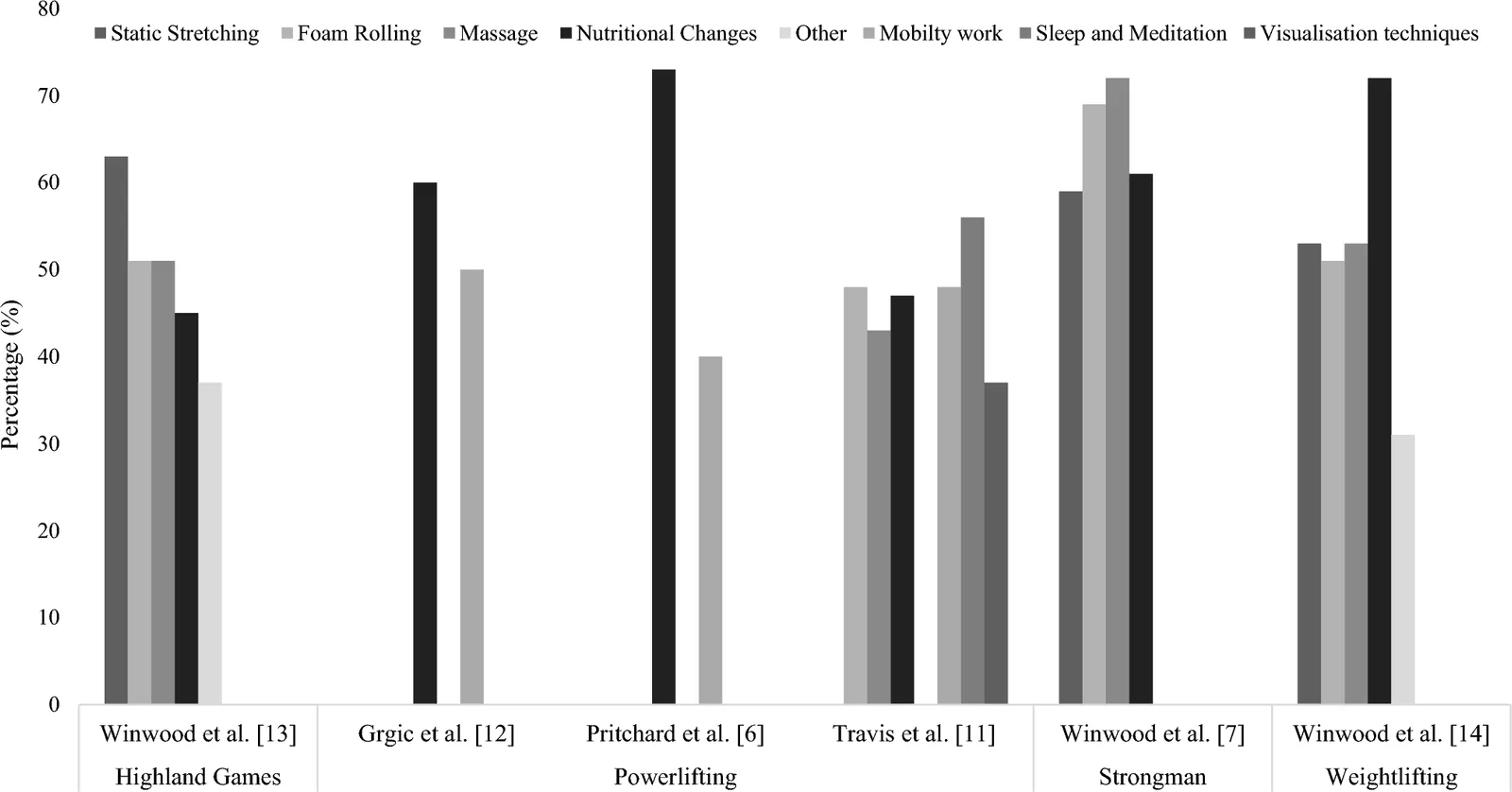
Other types of strategies utilized by Highland Games, powerlifting, strongman and weightlifting athletes during the taper

Across the weight lifting sports, athletes reported using a range of additional strategies during the taper, with nutrition related changes and recovery methods most consistently employed (see Fig. 4). Nutritional adjustments were among the most common taper strategies across sports, with the highest prevalence in powerlifting, strongman, and weightlifting (≈60–73%), while Highland Games heavy event athletes reported them less frequently (45%). Recovery-based approaches, such as massage, foam rolling, and static stretching, were widely reported, particularly among strongman (59–72%), Highland Games (51–63%), and weightlifting (51–53%) athletes, but were less frequently adopted in powerlifting. In contrast, psychological strategies appeared more distinctive to powerlifting, with over half of athletes reporting the use of sleep and meditation (56%) and more than a third employing visualization techniques (37%), strategies not reported in the other sports.

#### Qualitative Findings on Tapering

In addition to quantitative outcomes, several studies reported qualitative data describing athletes’ perceptions of tapering. These insights, derived from thematic analyses, provided context for why athletes choose to taper and why tapering may not have been effective.

#### Reasons for Tapering

Across the seven included studies, the most frequently reported reasons for tapering were physical recovery, peak performance, rest and psychological readiness. Physical recovery emerged as a universal theme, with athletes from CrossFit, Highland Games, powerlifting, strongman, and weightlifting all highlighting the need to reduce fatigue and muscle soreness before competition. Peak performance was the next most common theme, reported by athletes in CrossFit, Highland Games, strongman, and weightlifting, reflecting the intent to optimize physiological condition for competition. Rest and psychological readiness were both cited with equal frequency, particularly among CrossFit, strongman, and weightlifting athletes. Additional themes such as injury prevention, training specificity, and supercompensation were noted less frequently and appeared to reflect sport-specific tapering strategies.

#### Reasons for Ineffective Tapering

Across the included studies, athletes reported a range of reasons for ineffective tapering, with several recurring themes across weight lifting sports. The most commonly reported themes included tapering too long, training too close to competition at high intensity or volume, and external life or work stressors that disrupted recovery. In both weightlifting and strongman, athletes frequently cited training too hard or too light as a contributor to suboptimal competition performance. Poor planning, fatigue accumulation, and insufficient rest or sleep were also highlighted, particularly in strongman and Highland Games heavy event athletes. Additionally, athletes across all sports referenced nutrition related issues, such as weight cuts or dehydration, as contributing to poor taper outcomes. Unexpected factors like illness, travel issues, poor sleep environments, or changes in competition scheduling were also commonly noted.

## Discussion

This review provides the first integrated synthesis of tapering practices across the weight lifting sports, including CrossFit, Highland Games, powerlifting, strongman, and weightlifting. Tapering emerged as a near-universal strategy, typically short in duration (5–9 days) and characterized by step reductions in training volume of ~ 40–45%. Yet within this broad pattern, clear distinctions appeared by sport, sex, and athlete profile. Strongman and Highland Games competitors favored longer, step-based approaches, whereas weightlifters and CrossFit athletes more often reported shorter and sometimes linear tapers. Lift-specific patterns were also consistent: deadlifts were programmed furthest from competition, squats fell in an intermediate window, and bench press was maintained closest to meet day. Beyond load manipulation, athletes emphasized recovery, nutrition, and psychological readiness as integral to tapering, while ineffective strategies were most often linked to poor planning or external stressors such as work and injury. Overall, these findings accentuate both the universality of tapering across weight lifting sports and the sport-specific differences that necessitate tailored, evidence-based guidelines.

### Athlete Profiles across Weight Lifting Sports

This review highlights distinct demographic and experiential profiles across weight lifting sports that likely shape tapering practices. CrossFit and weightlifting athletes were generally younger (mid-to-late 20 s), lighter, and less experienced, while strongman and Highland Games heavy event athletes were older (30 s–40 s), heavier, and more experienced, with powerlifters occupying an intermediate profile (~ 28–29 years). Highland Games heavy event athletes stood out as the oldest (40.8 ± 10.7 years; 107 kg) [15], strongman athletes reported high body mass (108.6 ± 27.9 kg) and training experience [9], while CrossFit athletes averaged 26.5 ± 3.6 years and 74.5 ± 12.7 kg [10]. Weightlifters averaged 29.2 ± 8.7 years [16], and powerlifters across nations typically fell between these extremes [4, 13,14].

Force-dominant sports (i.e., powerlifting, strongman, Highland Games) often employ longer, step-based reductions, consistent with evidence that larger, stronger athletes may require more recovery [1, 3]. In contrast, power- and skill-oriented sports (i.e., weightlifting, CrossFit) favored shorter, sometimes linear approaches to preserve technical sharpness and neuromuscular readiness [10, 16]. Experimental findings support this distinction, showing that stronger athletes benefit more from extended load reductions, whereas weaker or less-trained athletes may not [13, 17]. When compared to the 2–4 week models typical of endurance and team sports [2, 18,19], weight lifting sports rely on shorter tapers of 5–9 days, balancing rapid recovery against the risk of detraining [20,21]. Collectively, these patterns demonstrate that tapering must be contextualized around athlete profile, sport demands, and the balance of recovery versus readiness before competition. Understanding these distinctions sets the stage for examining how training structures preceding the taper further shape strategy.

### Training Characteristics

Despite demographic differences, training structures were broadly similar across sports, with most athletes reporting four to five weekly sessions lasting 90–120 min. CrossFit athletes devoted the greatest proportion to conditioning, averaging four sessions weekly, while weightlifters emphasized technical practice of Olympic lifts with minimal dedicated conditioning (~ one session per week). Strongman and Highland Games heavy event athletes occupied the middle ground, combining two to three conditioning sessions with high event-specific loads and shorter overall durations via ~ 83–94 min. Powerlifters consistently trained four times weekly, with volume peaking 5–8 weeks before competition and intensity peaking ~ 2 weeks out.

Interestingly, although sex differences were limited, female Highland Games heavy event athletes consistently reported shorter sessions than males (74 ± 25 vs. 94 ± 38 min) [15]. Collectively, these patterns emphasize that while frequency and duration are broadly shared, sport-specific demands shape how training load is distributed. These demands also explain why taper strategies diverge from endurance models. Endurance athletes sustain adaptations with 2–4 week tapers, aided by slower-to-detraining aerobic mechanisms such as hematological and oxidative enzyme stability [21,22]. Short-term cessation studies demonstrate that maximal strength can be maintained, or briefly enhanced, after 2–6 days of rest, but upper-body strength declines faster than lower-body strength [23–25]. Bosquet et al. (2013) found via meta-analysis that strength, force, and power decline after resistance training cessation, with greater losses over longer inactivity. Consistent with this pattern, recent experimental work showed that 2–4 days of rest maintained performance, whereas longer cessation periods were associated with reductions in performance [23]. These findings may explain why shorter taper durations (5–9 days) are most effective in weight lifting sports: recovery is achieved quickly, but performance readiness may deteriorate if rest is prolonged.

Therefore, tapering cannot be separated from what precedes it. Power and skill-oriented sports (i.e., CrossFit, weightlifting) favor shorter, more gradual reductions to preserve technical sharpness, while force-dominant sports (i.e., strongman, Highland Games) employ slightly longer approaches to dissipate accumulated fatigue. However, powerlifting demonstrates both extremes, with most athletes tapering 7–10 days, but elite subgroups extending further to accommodate differences across the three maximal lifts [13]. Experimental comparisons reinforce this divide: step reductions of ~ 50% appear particularly effective for maintaining neuromuscular readiness in strength contexts [26], whereas endurance sports more often employ gradual exponential tapers to preserve oxidative fitness.

### Prevalence of Tapering

Tapering emerged as a near-universal practice across weight lifting sports. Adoption was highest in powerlifting, CrossFit, and weightlifting, where nearly every athlete reported tapering prior to competition, while prevalence was somewhat lower in strongman (87%) and Highland Games heavy event athletes (78%). Importantly, rates remained consistently high across competition level, age, and bodyweight, suggesting tapering is a foundational preparation strategy regardless of demographic subgroup. Notable differences did emerge, suggesting that strongman athletes and female competitors across sports reported the most consistent tapering, whereas Highland Games heavy event athletes and regional-level weightlifters were least consistent. In CrossFit, 98.6% of athletes tapered before competition, whereas in weightlifting, 99% reported tapering, with prevalence highest among coached and elite lifters. The powerlifting studies consistently showed > 95% taper adoption. In contrast, decentralized or tradition-based sports like strongman and Highland Games demonstrated greater variability. Collectively, these findings suggest that cultural and structural differences, particularly the presence or absence of formal coaching, may shape how consistently tapering is applied.

Unlike endurance sports, where tapering strategies were established through decades of experimental and observational research showing 2–4% performance gains following multi-week reductions [2, 18], tapering in weight lifting sports spread largely through experiential practice. Over the last decade, systematic research has confirmed the efficacy of tapering for strength and power purposes. Case studies in elite weightlifters [27,28], controlled experimental cessation studies [23–25], and experimental tapering studies [26, 29] demonstrate that reductions of ~ 40–50% in training load over 1–2 weeks within minimal pre-competition cessation reliably support strength performance [3, 30]. These data validate long-standing practice and highlight how athlete-led strategies preceded formal evidence.

Thus, the significance of this synthesis is twofold: tapering is now firmly embedded across weight lifting sports, and its application differs meaningfully from endurance models. Whereas endurance athletes typically employ gradual 2–4 week reductions, weight lifting athletes employ shorter (**≈**5–9 day) and sharper strategies designed to optimize physical recovery, peak performance, and psychological readiness. This review provides the first systematic framework tailored to these realities that integrates experiential practice with available empirical evidence.

### Taper Models

Across all weight lifting sports, step tapers were the most consistently reported approach. This format, characterized by an abrupt reduction in training load maintained until competition, appears to be preferred for its simplicity and reliability. Linear tapers, where load is gradually reduced, were more common in CrossFit and weightlifting, particularly among female athletes, while exponential models appeared in specific contexts, most notably in powerlifting. Strongman and Highland Games heavy event athletes overwhelmingly relied on step tapers (82–88%), consistent with their tradition-driven and decentralized coaching cultures. In contrast, Croatian powerlifters frequently adopted exponential or fast-decay models, whereas North American survey data confirmed step taper dominance (~ 70%) with some variation by sex and level.

Experimental studies further support the efficacy of implementing the step taper model. Pritchard et al. [24] found that a 7-day step taper reducing volume by ~ 70% improved maximal strength, while more recent work in strength-trained athletes has shown that ~ 50% reductions are also effective [26, 29]. These findings may help explain why step tapers dominate across weight lifting sports, whereas endurance athletes often rely on gradual exponential formats to preserve oxidative capacity [2, 19]. Together, the available evidence suggests that taper models may be influenced by cultural and structural factors, with step tapers commonly reported across weight lifting sports and linear or exponential approaches appearing more frequently in structured or coached environments.

### Taper Duration

Taper duration showed both consistency and divergence across weight lifting sports. CrossFit, Highland Games, strongman, and weightlifting athletes typically employ short tapers of ~ 5–9 days, balancing recovery with neuromuscular readiness. Powerlifting stood out as an outlier where interview-based studies in New Zealand and Croatia showed longer taper durations of 13–18 days, whereas larger North American survey data indicated most lifters tapered for only 7–10 days. This heterogeneity likely reflects differences in study design, athlete preferences, and the demands of managing three maximal lifts, as well as differences in international standing. U.S. and Canadian lifters, consistently ranked among the world’s top nations, appeared to favor shorter, sharper taper strategies compared to lifters from smaller competitive nations. Subgroup differences were modest but notable, demonstrating that strongman female athletes reported slightly longer tapers than men (9.5 ± 5.5 vs. 8.3 ± 4.8 days), while weightlifting men tapered longer than women (8.6 ± 4.6 vs. 5.3 ± 1.8 days). Competition level, age, and bodyweight rarely influenced taper length, except in powerlifting, where national-level lifters favored 7–10 day tapers and international lifters often used < 7 day windows.

Interestingly, experimental evidence reinforces the importance of taper length through controlled interventions. Pritchard et al. [31] implemented a 7-day taper and observed strength preservation, while Travis et al. [29] directly compared 1- vs. 3-week tapering, showing shorter durations improved muscular qualities and competition performance rather than laboratory performance. Similarly, Burke et al. [23] found that one-week tapers enhanced readiness, but longer rest impaired explosive performance. Collectively, these findings demonstrate the transient nature of neuromuscular adaptations via motor unit firing rates, neural drive, and acute hormonal responses that can peak within days and conversely deteriorate over time [29, 31,32]. Thus, short tapers (≤ 9 days) represent the prevailing standard across weight lifting sports, with powerlifting serving as the main exception, depending on country, level, and lifter profile.

### Adjustments in Training Volume, Intensity, and Emphasis

Across weight lifting sports, reductions in training volume during the taper clustered around ~ 40–45%. CrossFit athletes reported 41.2%, weightlifters 43%, and strongman 45%, whereas Highland Games heavy event athletes showed slightly smaller decreases (~ 34%), potentially reflecting the need to rehearse throwing events closer to competition. However, powerlifting displayed greater variability, from ~ 41% in North America to nearly 59% in New Zealand lifters, with Croatian champions averaging ~ 50%. These findings align with prior reviews [3, 30], which collectively suggest that performance peaks most reliably when training volume is reduced in the ~ 40–50% range, while intensity is typically maintained or slightly elevated. In the context of this review, training intensity refers to applied training measures (e.g., percentage of one repetition maximum (%1RM), movement velocity, or rate of perceived exertion (RPE)) as reported in the included studies.

Although intensity adjustments were less uniform, many athletes reduced intensity during the taper. However, the majority of powerlifters appeared to deliberately increase intensity, reflecting divergent philosophies and the challenge of peaking three maximal lifts. Importantly, Travis et al. [1] demonstrated that performance improvements can occur regardless of intensity being decreased, maintained, or increased, suggesting that volume reduction is the central driver of taper effectiveness. Experimental and observational work reinforces this point, where elite weightlifters have responded positively to ~ 50% volume reduction across three weeks [27,28], while strength-trained cohorts improved maximal strength following one-week tapers, cutting load by ~ 50% [26, 31]. Travis et al. [29] further highlighted that competition outcomes appeared to favor shorter step tapers, though longer exponential models sometimes optimized lab-based markers. Collectively, these findings show that sharper reductions with flexible intensity management align better with the rapid recovery and transient adaptations of weight lifting athletes than the prolonged reductions common in endurance contexts.

To promote recovery during the taper, there appeared to be a sport-specific emphasis further shaping taper execution. CrossFit athletes preserved conditioning and gymnastics alongside reduced strength training, Highland Games and strongman athletes emphasized strength-power with broader conditioning, and weightlifters focused almost exclusively on technical refinement and power. Conversely, powerlifters typically reduced or eliminated accessory work during the taper, channeling recovery and focus on the squat, bench press, and deadlift. These distinctions highlight that while tapering in weight lifting sports is structurally similar in magnitude, execution is dictated by the technical and physiological demands of each discipline, setting up the lift-specific taper strategies and timing considerations relative to the final days leading into competition.

### Timing of Training and Final Heavy Sessions

The timing of training cessation and final heavy sessions revealed both consistencies and lift-specific patterns. Rest periods clustered between ~ 1.5 and 4 days, with weightlifters reporting the shortest (1.5 ± 0.6 days), strongman the longest (3.9 ± 1.8 days), and powerlifters in between (2.8–3.7 days). Across sports, volume generally peaked 4–6 weeks out, while intensity peaked ~ 2–3 weeks before competition. CrossFit athletes showed slightly staggered peaks (i.e., conditioning ~ 4 weeks, strength ~ 5 weeks), whereas powerlifters displayed the widest range, with ~ 45% peaking 5–8 weeks out and ~ 30% within 4 weeks. International weightlifters and professionals across sports tended to peak further from competition, while local and regional athletes peaked closer.

Most interestingly, lift-specific tapering was highly repeatable. Squats were typically performed ~ 5–6 days before competition, bench press 2–4 days, and deadlifts 6–8 days, consistent with their recovery demands. Strongman extended squats slightly (~ 6.5 days), while Croatian and New Zealand champions clustered squat and deadlift 7–10 days out and bench closer (< 7 days). Additionally, North American lifters reported a similar hierarchy with deadlift furthest, bench closest, and squat intermediate. Relative loads were generally ~ 65–75% 1RM for squats and bench, though weightlifters often used ~ 80% squat loads and strongman favored heavier bench intensities at ~ 74%.

Interestingly, experimental findings do not support the notion that the deadlift induces greater fatigue or requires longer recovery than other compound lifts. Barnes et al. [33] observed similar acute neuromuscular and endocrine responses between squat and deadlift protocols, with no evidence of heightened central fatigue in the deadlift. Likewise, Belcher et al. [34] found comparable recovery trajectories across the squat, bench press, and deadlift, with no between lift differences in markers of muscle damage or performance recovery. Despite these findings, athlete surveys consistently report programming the deadlift furthest from competition, likely reflecting the greater absolute loads and systemic demands experienced in practice.

Experimental training cessation studies further corroborate these recovery windows. Pritchard et al. [24] found 3–5 days of cessation improved maximal strength, while longer breaks risked stagnation. Travis et al. [25] and Burke et al. [23] confirmed that lower-body performance could be preserved with 2–5 days of rest, while upper-body lifts benefited from slightly shorter cessation periods of 2–3 days. Case studies of elite weightlifters likewise showed that short periods of reduced or minimal training (≈2–5 days) supported competition readiness [27,28]. Collectively, these findings provide convergent support for lift-specific taper strategies, demonstrating that heavier, more systemically fatiguing lifts demand longer recovery windows than less taxing upper-body movements. However, additional recovery support may help potentiate further performance improvements during and after the taper.

### Coaching, Nutrition, Recovery, and Psychological Support

Support systems surrounding the athlete, such as coaching, nutrition, recovery, and psychological preparation, emerged as critical factors shaping taper implementation and effectiveness across weight lifting sports. Patterns of coaching involvement reflected structural and cultural differences. Weightlifting and CrossFit athletes most often reported formal coaching (73–75%), with female athletes particularly likely to receive structured guidance (87–94% vs. 61–63% of men). In contrast, self-coaching predominated in strongman (53%) and Highland Games (63%), especially among older, heavier, and professional athletes, where experience often substituted for formal oversight. Powerlifting displayed a mixed profile: Croatian and New Zealand lifters frequently cited coach-driven taper planning, whereas North American lifters were more likely to experiment or self-direct at the lower levels of competition. This divide suggests that sports embedded in structured systems (e.g., Olympic weightlifting, CrossFit, IPF-affiliated powerlifting) foster greater coaching input and diversity in taper models, while decentralized sports like strongman and Highland Games normalize tradition-driven approaches such as step tapers. Importantly, these cultural patterns echo a broader framework with Gee [35] suggesting that athletes’ ability to integrate psychological preparation, alongside technical and physiological input, is what ultimately brings them closer to their absolute performance potential, highlighting why formalized support systems may amplify taper effectiveness.

Beyond coaching, athletes emphasized nutrition, recovery, and psychological readiness as integral to tapering success. Nutritional strategies were sport-specific: powerlifters manipulated food intake primarily to meet weight-class requirements. This practice reflects broader trends reported in the literature, where powerlifters also emphasize peri-workout carbohydrate and protein timing to support recovery [36]. CrossFit athletes prioritized fueling adequacy across multi-event formats, often through structured diets and high supplement use. Similar approaches have been reported previously [33]. Weightlifters placed particular importance on carbohydrate availability and glycogen restoration. More broadly, nutritional interventions targeting recovery from exercise-induced muscle damage, such as protein and antioxidant intake, may further optimize taper outcomes [37]. Recovery approaches were similarly diverse with CrossFit and weightlifting athletes frequently employing structured modalities (i.e., mobility, massage, cryotherapy), whereas strongman and Highland Games heavy event athletes emphasized passive recovery and injury prevention. Powerlifters also incorporated stretching primarily for recovery and injury management rather than direct performance enhancement. This aligns with observations reported by Spence et al. [38].

Finally, psychological factors appeared to consistently shape taper effectiveness alongside physiological readiness. CrossFit athletes emphasized confidence and fatigue reduction, while powerlifters highlighted mental rehearsal and “feeling strong” on meet day. Recent work by Perkins et al. [39] demonstrated the importance of psychological monitoring during competition preparation with weightlifters, highlighting that correlations between biomarkers, performance outcomes, and subjective recovery-stress ratings can provide predictive insight into taper effectiveness. Importantly, peaking is not merely the sum of training load manipulations; rather, it reflects a synchronized system where physiological recovery, psychological readiness, and performance capacity converge. Supporting this, Rønnestad and Vikmoen [40] showed that athletes who achieved physiological improvements and improved subjective well-being during a compressed taper protocol realized superior performance gains compared to a traditional taper. These findings highlight that tapering failures can often be explained as much by mismatches in psychological preparedness as by training miscalculations. Thus, achieving a true peak requires aligning biological restoration, technical execution, and psychological arousal within the same narrow competition window.

### Weight Lifting Sports Tapering in the Context of Broader Literature

Early tapering frameworks emerged from endurance and team sports, where 2–4 week tapers with gradual 40–60% reductions in training volume consistently improved performance when intensity was maintained or slightly increased [2, 18]. In the absence of strength-specific evidence, barbell and hybrid athletes initially borrowed from these models, despite clear physiological differences between sustaining aerobic capacity and peaking maximal force and power production. The collective findings across the included studies suggest that direct extrapolation from endurance-based models may not be appropriate. Tapering in the weight lifting sports is characterized instead by shorter durations of 5–9 days, sharper step reductions in volume of ~ 40–50%, and lift-specific timing strategies, particularly extended recovery for the deadlift compared to the bench press. Experimental and observational work reinforces these distinctions, demonstrating that neuromuscular strength and power adaptations are transient and are susceptible to detraining and thus require brief, more precisely timed tapers [23–26, 29, 31].

This synthesis provides the first evidence-based alternative to endurance-derived models, offering discipline-specific guidelines that reflect the cultural, technical, and physiological realities of weight lifting athletes. By addressing the longstanding issue of “reverse borrowing,” it establishes a practical framework for future refinements in tapering strategies tailored to the unique demands of strength and power performance. While the current evidence base is limited to a small number of studies (*n* = 7), with some sports represented by only a single investigation, the consistency of findings across disciplines provides a useful foundation for understanding tapering practices in weight lifting sports.

## Conclusion

This review provides the first comprehensive synthesis of tapering practices across the weight lifting sports. Despite variation in athlete profiles, training structures, and competition formats, tapering emerged as a near universal strategy, typically short in duration (≈5–9 days) and characterized by ~ 40–50% reductions in training volume, with training intensity peaking two to three weeks before competition. Step tapers predominated, although higher level weightlifters and powerlifters also employed linear and exponential approaches. These patterns stand in contrast to the multi-week, gradual tapers typical of endurance sports, highlighting that strength disciplines generally favor shorter, sharper reductions in training load to balance recovery with competition readiness.

Sport specific distinctions were clear. Force-dominant athletes (e.g., Highland Games and strongman) tended to use longer, step based approaches, whereas power and skill oriented athletes (e.g., CrossFit and weightlifting) favored shorter or linear reductions. Lift sequencing was consistent across sports: deadlifts were placed furthest from competition, squats at an intermediate point, and bench press closest. Beyond load manipulation, nutrition, recovery modalities, and psychological preparation were central to taper effectiveness, while poor planning, external stressors, and nutritional mismanagement frequently undermined outcomes. Coaching support also differed, being most prevalent in weightlifting and CrossFit, and less common in strongman and Highland Games, where self-coaching predominated.

Collectively, these findings establish a discipline specific framework for tapering in weight lifting sports, bridging experiential practice with emerging empirical validation. They underscore the need for tailored tapering strategies that reflect cultural, technical, and physiological demands, rather than wholesale adoption of endurance-based models. Future research should expand controlled investigations across strength disciplines, integrate physiological and psychological monitoring, and refine individualized tapering frameworks to maximize competition readiness.

## Data Availability

The data supporting the findings of this study, including extracted datasets and PRISMA materials, are publicly available on the Open Science Framework at: https://osf.io/cu6yv/overview.
